# Characterization of the *Corynebacterium glutamicum* dehydroshikimate dehydratase QsuB and its potential for microbial production of protocatechuic acid

**DOI:** 10.1371/journal.pone.0231560

**Published:** 2020-08-21

**Authors:** Ekaterina A. Shmonova, Olga V. Voloshina, Maksim V. Ovsienko, Sergey V. Smirnov, Dmitry E. Nolde, Vera G. Doroshenko

**Affiliations:** 1 Ajinomoto-Genetika Research Institute, Moscow, Russian Federation; 2 Shemyakin–Ovchinnikov Institute of Bioorganic Chemistry RAS, Moscow, Russian Federation; Northern Arizona University, UNITED STATES

## Abstract

The dehydroshikimate dehydratase (DSD) from *Corynebacterium glutamicum* encoded by the *qsuB* gene is related to the previously described QuiC1 protein (39.9% identity) from *Pseudomonas putida*. Both QuiC1 and QsuB are two-domain bacterial DSDs. The N-terminal domain provides dehydratase activity, while the C-terminal domain has sequence identity with 4-hydroxyphenylpyruvate dioxygenase. Here, the QsuB protein and its N-terminal domain (N-QsuB) were expressed in the T7 system, purified and characterized. QsuB was present mainly in octameric form (60%), while N-QsuB had a predominantly monomeric structure (80%) in aqueous buffer. Both proteins possessed DSD activity with one of the following cofactors (listed in the order of decreasing activity): Co^2+^, Mg^2+^, Mn^2+^. The K_*m*_ and k_*cat*_ values for the QsuB enzyme (K_*m*_ ~ 1 mM, k_*cat*_ ~ 61 s^-1^) were two and three times higher than those for N-QsuB. 3,4-DHBA inhibited QsuB (K_i_ ~ 0.38 mM, K_i_’ ~ 0.96 mM) and N-QsuB (K_i_ ~ 0.69 mM) enzymes via mixed and noncompetitive inhibition mechanism, respectively. *E*. *coli* MG1655Δ*aroE*P_*lac*_‒*qsuB* strain produced three times more 3,4-DHBA from glucose in test tube fermentation than the MG1655Δ*aroE*P_*lac*_‒*n*-*qsuB* strain. The C-terminal domain activity towards 3,4-DHBA was not established *in vitro*. This domain was proposed to promote protein oligomerization for maintaining structural stability of the enzyme. The dimer formation of QsuB protein was more predictable (ΔG = ‒15.8 kcal/mol) than the dimerization of its truncated version N-QsuB (ΔG = ‒0.4 kcal/mol).

## Introduction

**Fig 1:**
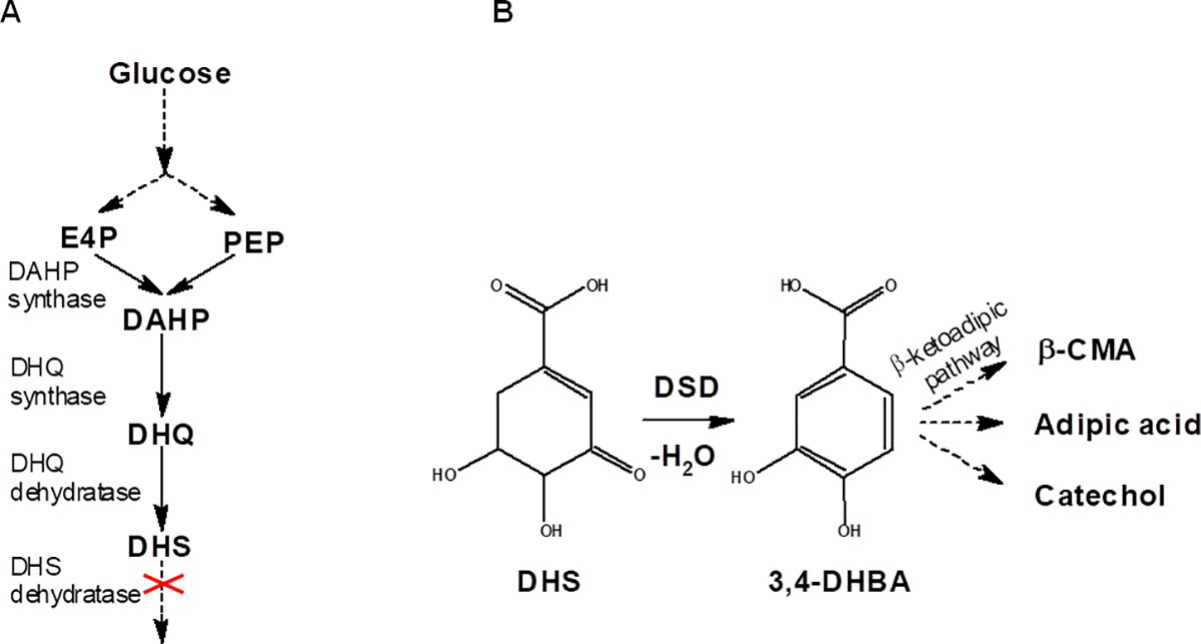
Biosynthesis of 3,4-DHBA from 3-dehydroshikimic acid (DHS), an intermediate of the common aromatic pathway. (A) Synthesis of DHS from glucose. (B) Formation of 3,4-DHBA from DHS with the help of DSD. Abbreviations: β-CMA - β-carboxy-*cis*,*cis*-muconic acid; DAHP—3-deoxy-D-arabino-heptulosonate-7-phosphate; DHQ—3-dehydroquinate; E4P - erythrose 4-phosphate; PEP—phosphoenolpyruvate.

Protocatechuic acid or 3,4-dihydroxybenzoic acid (3,4-DHBA) is a catechol-type phenol present in some fruits and vegetables [1, 2]. It has been shown that 3,4-DHBA displays antibacterial [3], antimutagenic [4], anti-inflammatory [5], antihyperglycemic [6] and highly antioxidant [7] effects. It also has potential applications for further microbiological synthesis of numerous valuable compounds, including the bioplastic precursor *cis*,*cis*-muconic acid [8, 9]. To produce 3,4-DHBA from glucose, the synthesis of this compound from DHS, an intermediate in the common aromatic pathway, was previously implemented in *E*. *coli* cells [10, 11]. This reaction was catalyzed by dehydroshikimate dehydratase (DSD) (EC: 4.2.1.118) (Fig 1), a part of the quinate and shikimate degradation pathways in soil-dwelling bacteria and fungi. Another source of this enzyme is the biosynthesis of the unique 3,4-catecholate moiety found as part of the petrobactin scaffold present in the *Bacillus cereus* group. DSD is encoded by the *asbF* gene, being identical in *Bacillus anthracis* and *Bacillus thuringiensis*, and has been thoroughly investigated [12‒14]. Based on primary structure analysis, all known DSDs were divided into four groups: fungal single-domain, bacterial two-domain, AsbF-like, and bacterial membrane-associated enzymes [15]. The application of AsbF and fungal (*Podospora pauciseta*) and bacterial two-domain (*Klebsiella pneumoniae*) enzymes to obtain 3,4-DHBA as an intermediate for the generation of valuable compounds from glucose has been demonstrated [10, 11, 16, 17].

The well-known industrial microorganism *Corynebacterium glutamicum*, which has generally recognized as safe (GRAS) status, harbors DSD but the enzyme has not yet been characterized biochemically. DSD is encoded by the *qsuB* gene, a part of the operon *qsuABCD*, whose products are involved in the quinate/shikimate utilization pathway [18]. Previously, QsuB of *C*. *glutamicum* was assigned to the two-domain bacterial DSD group [15]. QuiC1 from *Pseudomonas putida* was the only characterized member of this class. The N-terminal domain of QuiC1 catalyzes a dehydratase reaction, while the C-terminal domain has significant sequence identity with 4-hydroxyphenylpyruvate dioxygenase (HPPD) (EC: 1.13.11.27). The role of the C-terminal domain is still unknown, but it was suggested that it might be involved in 3,4-DHBA degradation via the ß-ketoadipate pathway [15]. Here, we examined the biochemical characteristics of full-length QsuB and its N-terminal domain (N-QsuB) as well as QsuB application to 3,4-DHBA production in heterologous host.

## Materials and methods

### Bacterial strains and growth conditions

Known laboratory *E*. *coli* strains [18]: MG1655 (F^‒^λ^‒^*ilvG rfb*-50 *rph*-1) and BL21(DE3) (F^–^*ompT gal dcm lon hsdS*_*B*_(r_B_^–^m_B_^–^) λ(DE3[*lacI lacUV5-T7p07 ind1 sam7 nin5*]) [*malB*^*+*^]_K-12_(λ^S^) were subjected to further modifications in this work. The strains were cultivated in rich LB medium [19]. For the preparation of electrocompetent *E*. *coli* cells, SOB medium was used [20]. Antibiotics, when required, were added in the following concentrations (mg/l): ampicillin– 200, chloramphenicol– 20, tetracycline– 12.5.

Cell cultures for the isolation of the QsuB and N-QsuB proteins were prepared as follows. Flasks containing 30 ml LB with ampicillin were inoculated with overnight cultures (300 μl) of the BL21(DE3)/pET22b-*qsuB* and BL21(DE3)/pET22b-*n*-*qsuB* strains. The flasks were incubated at 25°C and 200 rpm for 2 h, then subjected to 1 mM isopropyl β-D-1-thiogalactopyranoside (IPTG) induction and incubated for an additional 20 h.

Fermentations were performed in tubes (18 x 200 mm) containing 2 ml of the production medium: 40 g/l glucose, 60 g/l CaCO_3_, 10 g/l tryptone, 10 g/l NaCl, 5 g/l yeast extract, 0.5 g/l (NH_4_)_2_SO_4_, 0.5 g/l K_2_HPO_4_, 5 mg/l FeSO_4_ x 7H_2_O, 4 mg/l MnSO_4_ x 5H_2_O, 10 mg/l thiamine, 10 mg/l 4-hydroxybenzoic acid, 10 mg/l 4-aminobenzoic acid, and 10 mg/l 2,3-dihydroxybenzoic acid. The fermentation medium was prepared by mixing sterile components. Then it was divided into parts which was supplemented with IPTG (1 mM) and CoCl_2_ (10 μM, 100 μM) or not. The fermentation tubes were inoculated with 0.2 ml of seed culture. To prepare seed culture, one loop (3 mm) of cells from a fresh plate was inoculated into a tube (13 x 150 mm) containing 3 ml of LB and incubated at 34°C with aeration (240 rpm) for 3 h. The fermentation tubes were cultivated at 34°C (250 rpm) for 44 h. After that culture broth was diluted to determine OD and product concentrations.

### DNA manipulation

All recombinant DNA manipulation was conducted according to standard procedures [19] and the recommendations of the enzyme manufacturer (Thermo Scientific, USA). Plasmid and chromosomal DNAs were isolated with Plasmid Miniprep (Evrogen, Russia) and PurElute Bacterial Genomic Kit (Edge BioSystems, USA), respectively. PCR was performed with Taq DNA polymerase (GBM, Russia) and with Phusion DNA Polymerase (Thermo Scientific) for circular polymerase extension cloning (CPEC) [21]. Primers (S1 Table) were purchased from Evrogen. Plasmids and genetic modifications of the *E*. *coli* chromosome were verified by sequence analysis.

### Plasmid construction

The plasmids pET22b-*qsuB* and pET22b-*n*-*qsuB* were obtained by CPEC followed by selection in BL21(DE3) cells. The DNA fragment containing pET22b (Novagen, USA) was amplified using the primers P1/P2. The DNA fragments of *qsuB* and *n*-*qsuB* were amplified using the chromosomal DNA of *C*. *glutamicum* 2256 ATCC13869 as a template and the primers P3/P4 and P3/P5, respectively.

To integrate the *qsuB* gene into the *E*. *coli* chromosome, a DNA fragment containing P_*lacUV5*_-*qsuB* was cloned into the integrative vector pAH162-λ*attL*-*tet*-λ*attR* [22] between the *Sal*I and *Sac*I restriction sites. The DNA fragment containing P_*lacUV5*_-*qsuB* was obtained by overlapping PCR (primers P6/P9) of DNA fragments containing the promoter P_*lacUV5*_ and the *qsuB* coding region. These DNA fragments were amplified by PCR with the primers P6/P7 and P8/P9 using pELAC [23] and pET22b-*qsuB* plasmid DNA as templates, respectively.

### Strain construction

To construct the MG1655Δ*aroE* strain, an in-frame deletion of the *aroE* gene was created using λRed-mediated integration of a DNA fragment containing the excisable marker λ*attL*-*cat*-λ*attR* (primers P10, P11). The recombinant plasmids pKD46 and pMW118-λ*attL*-*cat*-λ*attR* were used as a helper for the λRed-mediated integration and as a template for the excisable marker λ*attL*-*cat*-λ*attR*, respectively [20, 24]. The marker was excised using the helper plasmid pMW-*int*-*xis* [24].

The MG1655Δ*aroE*P_*lac*_-*qsuB* strain was created via integration of P_*lacUV5*_-*qsuB* into the native φ80-*attB* site of the MG1655 chromosome. The integrative vector pAH162-λ*attL*-*tet*-λ*attR* with the cloned modification P_*lacUV5*_-*qsuB* (see above) containing the φ80-*attP*-site and the pAH123-helper plasmid were used [22].The MG1655Δ*aroE*P_*lac*_-*n*-*qsuB* strain was generated via λRed integration of the excisable marker λ*attL*-*cat*-λ*attR* instead of the 3’-part of the *qsuB* coding region (primers P12/P13) into the chromosome of the MG1655Δ*aroE*P_*lac*_-*qsuB* strain, followed by marker removal.

### Obtaining crude extracts and purified proteins

All manipulations were performed at 4°C. After cultivation, cells were collected by centrifugation at 13200 rpm for 5 min and washed twice with 0.9% NaCl. The pellets were resuspended in 0.5 ml of 0.1 M potassium phosphate (pH 7.5), 0.1 mM EDTA, and 0.4 mM dithiothreitol buffer solution with the addition of 0.1 mM phenylmethylsulfonyl fluoride. The obtained suspensions were sonicated and then centrifuged at 13200 rpm for 5 min. The supernatants were decanted and then subjected to enzymatic reactions and 12% SDS-PAGE. PageRuler Prestained Protein Ladder 26616 (Thermo Scientific) was used to evaluate protein molecular mass.

For protein purification, the cells were resuspended in 15 ml of the abovementioned buffer and disrupted with a French press. The cell debris was eliminated by centrifugation at 6000 rpm for 5 min. Then, the supernatant was applied to Ni-NTA Affinity Resin (Clontech, USA). The protein fractions were eluted with an imidazole gradient from 20 mM (in the binding buffer) to 500 mM (in the elution buffer). The fraction containing the purified protein was dissolved in a buffer with 50 mM Tris-HCl (pH 7.5), 500 mM NaCl and 5% glycerol.

### Gel filtration

Molecular mass determination was carried out using gel-filtration chromatography with a Superose 6 Increase 10/300 GL (GE Healthcare, USA) column. A buffer solution containing 50 mM sodium phosphate (pH 7.0), 150 mM NaCl and a flow rate of 0.5 ml/min was used for N-QsuB. A buffer solution containing 50 mM Tris-HCl (pH 7.5), 500 mM NaCl, and 5% v/v glycerol and a flow rate of 0.4 ml/min were used for QsuB. Proteins were detected by monitoring the absorbance at 280 nm. The gel filtration marker kits MWGF 1000 (29 ‒700 KDa) and MWGF 70 (12.4 KDa) were purchased from Sigma-Aldrich, USA.

### Enzyme activity assays

The DSD activity of QsuB was initially detected using the purified recombinant protein as well its shortened version N-QsuB. The enzymes (10‒100 nM) were incubated in 1 mL cuvette with 0.1 M Tris/HCl buffer (pH 7.5), 10 mM MgCl_2_, 1 mM dehydroshikimate for 1–5 min. Product identification was performed via comparison of its UV spectrum with that of 3,4-DHBA standard using Genesys10S UV-visible spectrophotometer (Thermo Scientific) (Fig A in S1 File). The identity of the compounds to DHS and 3,4-DHBA standards was verified using HPLC (Fig B in S1 File). For this purpose, the reaction was quenched by adding ethanol up to concentration of 70%, diluted by 100-fold in water and filtrated. The compounds from samples of the reaction mixture were separated by HPLC (Shimadzu Prominence) with diode array detector SPD-M20A (Shimadzu, USA) equipped with Zorbax eclipse column (XDB-c18; 3,0x150mm, 3,5μm) (Agilent Technologies, USA) at 30°C. Mobile phase consisted of 4 mM H_2_SO_4_ and methanol (90%, v/v). Methanol gradient (20% - 7 min, 35% - 3 min, 50% - 3 min, 20% - 5 min) at a flow rate of 0.25 ml/min was used. UV detection was at 235 nm for DHS and 260 nm for 3,4-DHBA. DHS and 3,4-DHBA standards were purchased from Sigma-Aldrich, USA.

The kinetic properties of QsuB and N-QsuB enzymes were measured by following the production of 3,4-DHBA (ε_290_ = 3.89*10^3^ M^-1^ cm^-1^) at 290 nm using the described procedure [25]. The reaction rate was determined from a linear fit to the change in absorption. The reaction mixtures were prepared in 1 ml volumes and monitored at 20°C for 1 min. Typical reactions contained 20 nM (QsuB) or 50 nM (N-QsuB) purified protein, 0.1 M Tris/HCl buffer (pH 7.5), 0.1 ‒ 5 mM DHS and 10 mM metal salt. To remove divalent cations from the enzyme preparation, the enzyme mixture was incubated in the presence of 1 mM EDTA on ice for 1 h. The metal cofactors were determined by monitoring the reaction in the presence of 1 mM DHS and each of tested metal salts: CaCl_2_, CoCl_2_, MgCl_2_, MnCl_2_, and ZnSO_4_. pH profiles were obtained in the presence of 1 mM DHS and 10 mM CoCl_2_.

V_max_ and K_m_ were obtained by plotting the graph in double reciprocal coordinates.

An inhibition type and inhibition constants of the enzyme reaction were determined by a graphical method [26]. The experimental data were plotted as (V_max_–v)/v versus the inhibitor concentration (0 ‒ 0.4 mM 3,4-DHBA) at 0.5/ 1/ 1.5 mM concentrations of DHS, where V_max_ and v represented the maximal velocity and the velocity in the absence and presence of inhibitor with given concentrations of the substrate, respectively. The dioxygenase activity of the C-terminal domain was tested with the crude extracts in the reaction mixture of 0.1 M Tris/HCl buffer (pH 7.5), 10 mM MgCl_2_, 0.1 mM DHBA, and 30 nM of overall protein.

### Statistical analysis

All values in graphs and tables are presented as arithmetic means of at least three independent experiments. Given errors are standard deviations. Microsoft Excel 2010 was used for calculations.

### Sequence alignment and 3D structural analysis

The QsuB sequence was aligned with QuiC1 of *P*. *putida* using T-Coffee software [27]. The 3D structure of QsuB was predicted using I-TASSER software [28]. Protein model of *P*. *putida* DSD QuiC1 (PDB code 5HMQ) was downloaded from Protein Data Bank (http://www.rcsb.org) [29]. Dimers of QsuB and N-QsuB proteins were modeled based on the QuiC1 dimer using the PyMOL Molecular Graphics System (ver. 1. 2r3pre, Schrödinger, LLC). Dimer formation was analyzed using PISA service (https://www.ebi.ac.uk/msd-srv/prot_int/pistart.html) [30].

## Results

### Recombinant expression and oligomeric state determination of QsuB and its DSD domain

**Fig 2:**
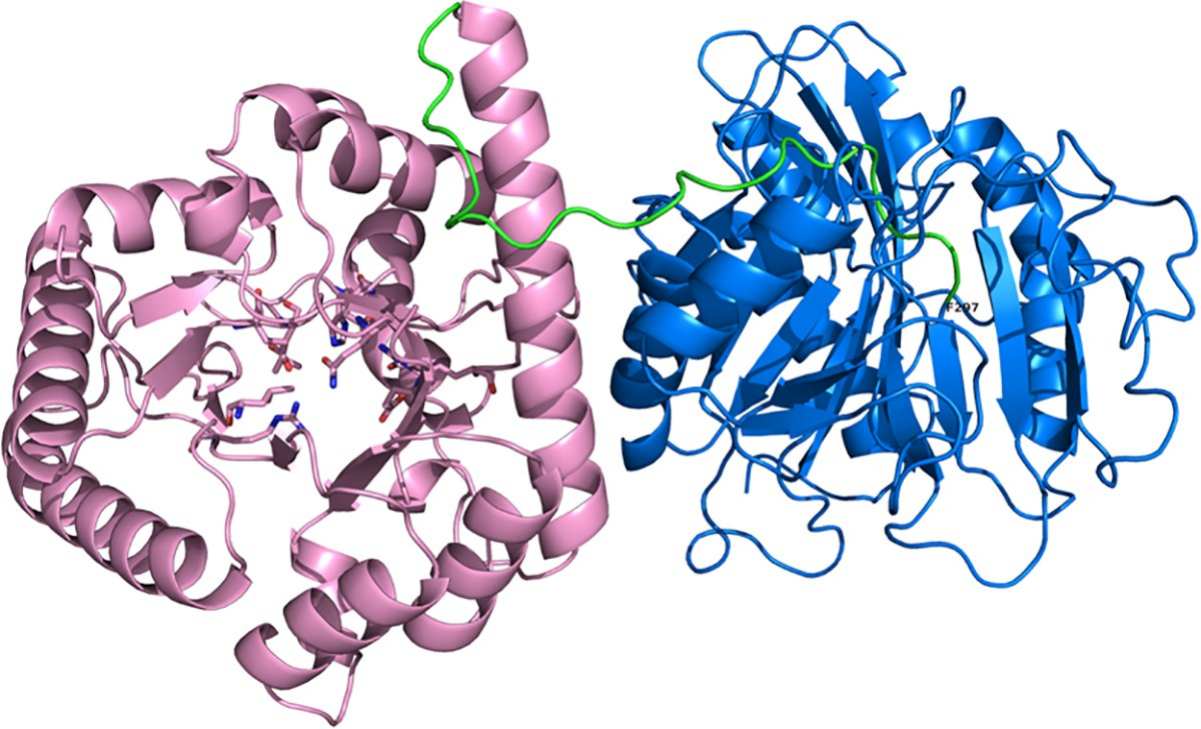
Predicted structure of the QsuB monomer. N, C-terminal domains and linker are shown in pink, blue and green colors, respectively. The residues of active center of DSD are represented as sticks. The terminal residue Phe297 of N-QsuB protein is indicated.

According to the protein sequence alignment, QsuB had 39.9% identity with QuiC1 of *P*. *putida* and 50.9% identity in the N-terminal domain coding for DSD activity. This level of identity allowed us to predict the 3D structure of QsuB based on the QuiC1 crystal structure (PDB ID: 5HMQ) (Fig 2). All the residues identified previously in the active site of DSD of QuiC1 [15] were also present in the N-terminal domain (1 ‒ 276 amino acids) of QsuB (Arg64, Leu97, Glu134, Leu136, His168, Asp165, Gln191, Ser206, Arg210 and Glu239).

**Fig 3:**
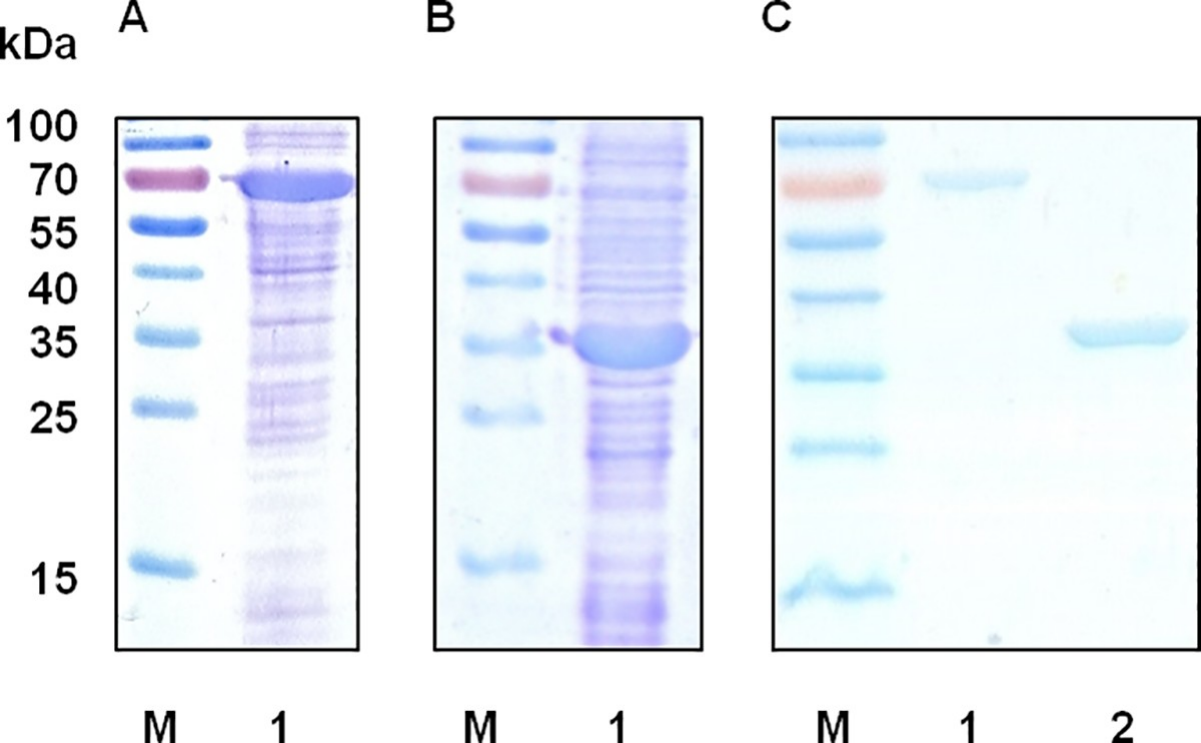
Expression of QsuB and N-QsuB in the T7 system. Extracts of cells after IPTG induction were analyzed using 12% SDS-PAGE. M, protein marker. (A) Lane 1, crude extract of BL21(DE3)/pET22b-*qsuB* cells. (B) Lane 1, crude extract of BL21(DE3)/pET22b-*n*-*qsuB* cells. (C) Proteins after purification: lane 1, QsuB (~ 70 KDa); lane 2, N-QsuB (~ 34 KDa). Raw images used to generate the figure are in S1 Raw images.

To investigate the importance of both domains in 3,4-DHBA synthesis, QsuB and its N-terminal domain (N-QsuB) were expressed in *E*. *coli* BL21(DE3) cells. The full-length protein and its truncated variant were clearly visible on the electropherograms of the cell extract proteins, where they accounted for approximately 15% of the total cellular proteins (Fig 3).

**Fig 4:**
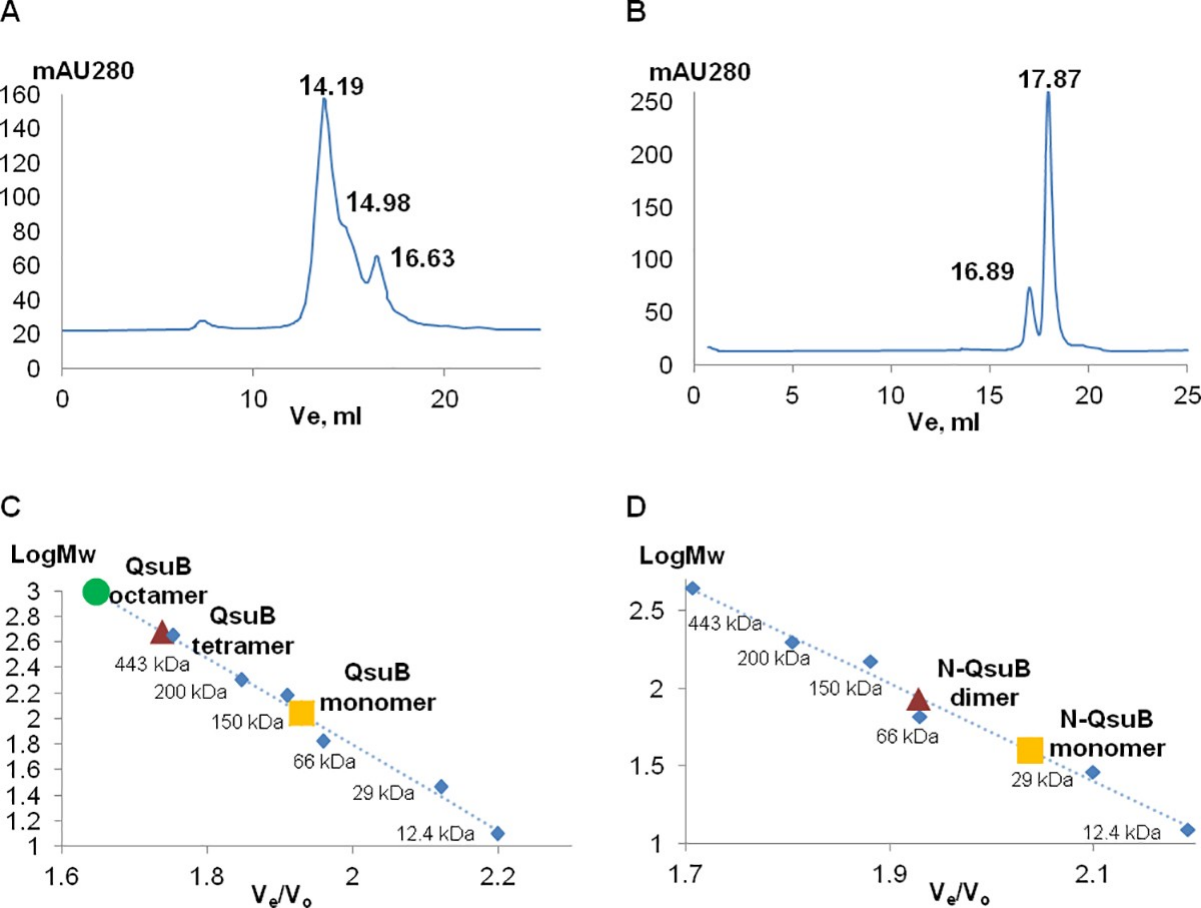
Oligomeric state determination of QsuB and N-QsuB. Molecular mass determination was carried out using SEC. Proteins were detected by monitoring absorbance at 280 nm. (A) Gel-filtration analysis of QsuB: the volumes of elution (V_e_) for the monomeric, tetrameric and octameric forms were 16.63 ml, 14.98 ml and 14.19 ml, respectively. (B) Gel-filtration analysis of N-QsuB: the V_e_ values of the monomeric and dimeric forms were 16.89 ml and 17.87 ml, respectively. The molecular mass calibration curves for QsuB (C) and N-QsuB (D) represent A_280 nm_, which is dependent on V_e_/V_o_, where V_0_ was the void volume of the column. V_0_ was determined experimentally as the V_e_ of Blue Dextran (2000 KDa). The standard proteins are represented with blue rhombuses and are cytochrome с (12.4 KDa), carbonic anhydrase (29 KDa), albumin (66 KDa), alcohol dehydrogenase (150 KDa), β-amylase (200 KDa), apoferritin (443 KDa) and thyroglobulin (669 KDa).

The purified His-tag proteins (Fig 3C) were used in gel-filtration experiments (Fig 4A and 4B). Analysis by size exclusion chromatography (SEC) indicated that QsuB tended to form multimeric structures: it occurred as 60% octamers, 25% tetramers and 15% monomers in aqueous buffer (Fig 4C). N-QsuB was present mainly in monomeric form (80%) (Fig 4D). Thus, the C-terminal domain could stimulate the formation of oligomeric structures.

### The DSD activity of QsuB: Effects of metal ions and pH

**Fig 5:**
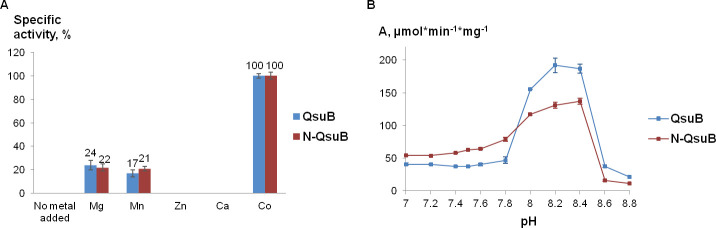
Effect of metal ions and pH on QsuB and N-QsuB DSD activity (1 mM DHS). (A) Relative DSD activities of QsuB and N-QsuB in the presence of divalent metals normalized against QsuB and N-QsuB in the presence of cobalt. (B) Dependence of QsuB and N-QsuB DSD activity on pH.

To analyze the *in vitro* DSD activity, QsuB and N-QsuB protein fractions collected after SEC were used. Both QsuB and N-QsuB stimulated the conversion of DHS to 3,4-DHBA that was assayed spectrophotometrically by monitoring the adsorption changing at 290 nm. The identity of the *in vitro* reaction product to 3,4-DHBA was confirmed using HPLC (Fig B in S1 File). As it was known from previous studies, DSDs had a requirement for divalent cations [12, 13]. Initially, QsuB and N-QsuB enzymes catalyzed the reactions without addition of metal ions, but these activities were inhibited by EDTA. Supplementation of the reaction mixture with Mg^2+^ restored the DSD activity. When studying the specificity to metal cofactors, we took into account the data of Fox et al. [12], who showed that iron and copper changed the spectrum at 290 nm in the presence of DHS without the addition of the AsbF protein. These metal ions were excluded from consideration. The enzymes were tested for catalytic activity in the presence of Ca^2+^, Co^2+^, Mg^2+^, Mn^2+^ and Zn^2+^. As shown in Fig 5A, Co^2+^ provided the maximal activity, followed by Mg^2+^ and Mn^2+^.

The pH dependence of QsuB and N-QsuB was investigated in the presence of Co^2+^ (Fig 5B). The optimum pH for these enzymes was 8.0 ‒ 8.4. AsbF enzyme also had optimal pH in alkaline range [12, 14]. At physiological pH 7 ‒ 7.8 for *C*. *glutamicum* and *E*. *coli* [31] the activities of both enzymes decreased, but N-QsuB demonstrated higher DSD activity at used saturated concentration of DHS.

### Kinetic analysis of QsuB and N-QsuB

**Fig 6:**
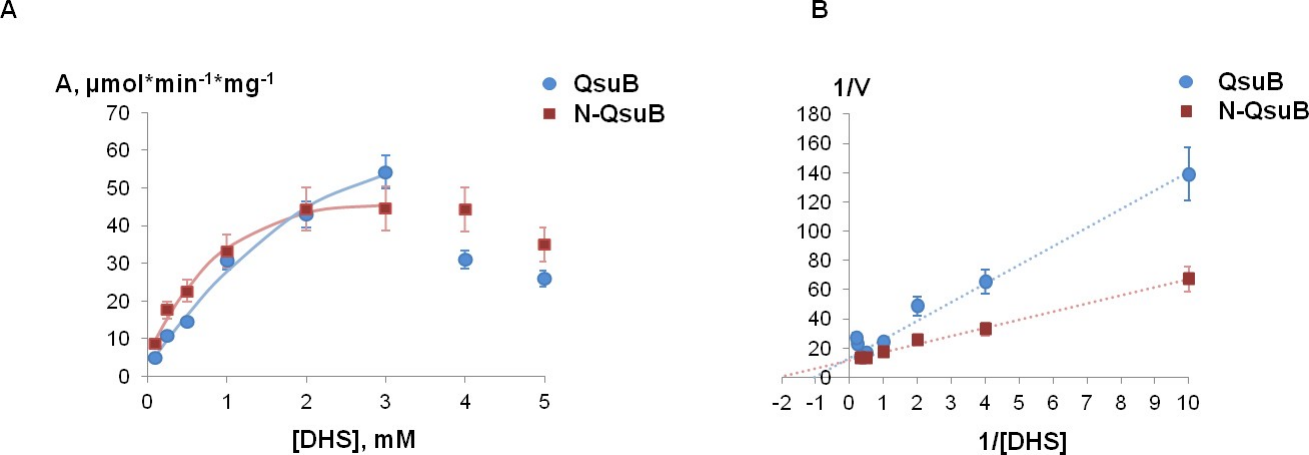
Kinetic curves of QsuB and N-QsuB. (A) Dependence of protein (20 nM QsuB, 50 nM N-QsuB) activity on substrate concentration. (B) Double reciprocal plots of protein kinetics.

Kinetic curves were obtained for QsuB and N-QsuB at pH 7.5 (Fig 6A), then K_*m*_ and k_*cat*_ parameters were calculated (Fig 6B; Table 1). As seen from Table 1, the removal of the C-terminal domain in QsuB decreased K_m_ and k_cat_ values by two and three times, respectively, with no significant change in K_eff_.

**Table 1:** Kinetic properties of QsuB and N-QsuB.

Protein	K_*m*_ (DHS), μM	k_*cat*_, s^-1^	k_*cat*_ /K_*m*_, (10^3^*μM^-1^s^-1^)	Source
QsuB	961 ± 77	60.8± 0.9	63.3 ± 11.7	This work
N-QsuB	473 ± 71	23.4 ± 0.4	49.5 ± 5.6	This work
QuiC1	331 ± 51	163.6 ± 8.5	494.3 ± 16.7	[15]
AsbF_*B*. *t*. 97–27_*	125 ± 14	3.6 ± 0.2	28.9± 11.9	[12]

*Kinetic properties of QsuB, N-QsuB and QuiC1 were determined at pH 7.5, 20°C and 25°C, respectively, while that of AsbF were determined at pH 8.6 and 37°C.

Having reached the maximum at 3 mM DHS, the DSD activity of QsuB and N-QsuB began to decrease. DHS adsorption at concentrations above 4 mM has already begun to affect the measurements accuracy of 3,4-DHBA formation. To confirm a decrease in the reaction rate observed above 3 ‒ 4 mM DHS, the product and substrate concentrations in the reaction mixture were verified by HPLC. Indeed, DHS decreased DSD activities of QsuB and N-QsuB enzymes with IC_50_-values ∼4.2 mM and ∼5.5mM, respectively. These values were calculated based on data from Fig 6.

**Fig 7:**
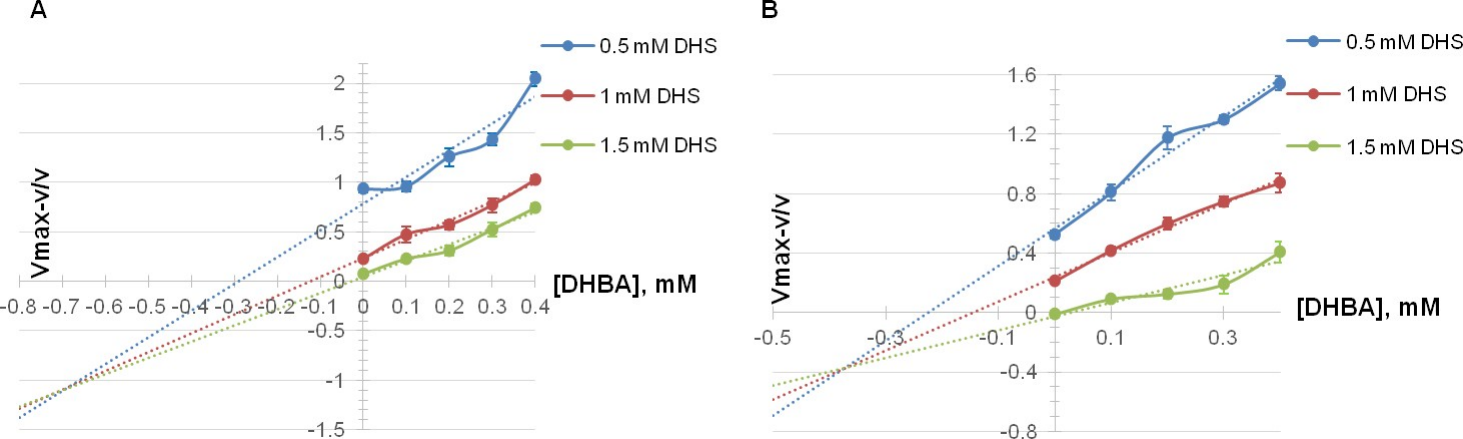
Quotient velocity plots demonstrating N-QsuB (A) and QsuB (B) inhibition. Different 3,4-DHBA concentrations are represented by colors: 0 (blue line), 0.3 (red line), and 0.6 mM (green line).

QsuB and N-QsuB were inhibited by 3,4-DHBA, and this inhibition was analyzed by the “quotient velocity plot” method [26] (Fig 7). As seen from Fig 7A, inhibition curves for N-QsuB obtained with different substrate concentration converged at–K_i_ = −1 that corresponded to noncompetitive inhibition (K_i_ ~ 0.69 mM). Whereas the same curves for QsuB matched to mix inhibition type with (K_i_ ~ 0.38 mM, K_i_’ ~ 0.96 mM). In the case of N-QsuB, the product could bind to the enzyme (K_i_), and in the case of QsuB, it could bind to both the enzyme (K_i_) and the enzyme-substrate complex (K_i_’). Thus, the removal of the C-terminal domain resulted in the elimination of enzyme-substrate complex inhibition by 3,4-DHBA.

### Investigation of C-terminal domain function

3,4-DHBA normally undergoes a dioxygenase-catalyzed ring cleavage during its degradation via ß-ketoadipate pathway. The corresponding enzymes are coded by *pca* gene cluster in *C*. *glutamicum* [32]. Followed to Peek et al. [15] we suggested that the C-terminal domain of QsuB could degrade 3,4-DHBA by the help of 3,4-DHBA dioxygenase activity.

**Fig 8:**
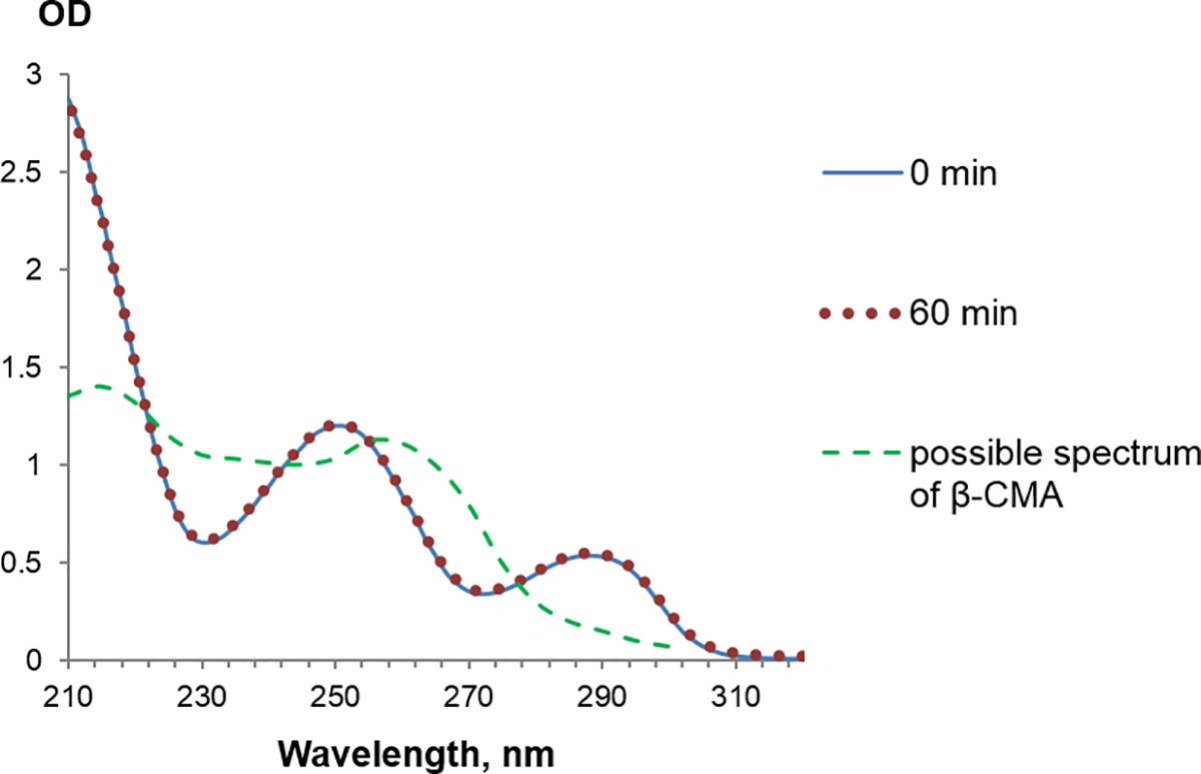
Stability of 3,4-DHBA in the presence of QsuB. The UV spectrum of the reaction mixture (pH 7.5) containing purified QsuB before and after 60 min incubation with 3,4-DHBA was the same and coincided with the 3,4-DHBA spectrum. The UV spectrum of β-CMA is shown in [33].

To check this suggestion, QsuB was incubated at pH 7.5 in the presence of 3,4-DHBA for up to 60 min, but no difference in the UV spectrum was observed (Fig 8). Thus, a possible activity of the C-terminal domain leading to conversion of 3,4-DHBA into another product was not detected under our experimental conditions.

As proposed above, C-terminal domain could participate in the oligomerization. The QuiC1 protein, also had a multimeric structure that was present as hexamer consisting of three dimers [15]. Assuming that the tetra- and octamers of QsuB protein could be formed from dimers, according to its similarity with QuiC1, the dimers of QsuB and N-QsuB proteins were analyzed. Dimer formation of QsuB was more likely (ΔG = ‒15.8 kcal/mol) than that of N-QsuB (ΔG = ‒0.4 kcal/mol). Contact interaction between QsuB monomers could be due to the formation of 12 hydrogen bonds and 4 salt bridges between residuals of N and C-terminal domains (data in S2 File). After removal of the C-terminal domain, dimerization could occur as a result of formation of 2 hydrogen bonds and 2 salt bridges between residuals of N-terminal domain (data in S3 File). Therefore, the dimers of N-QsuB could be extremely unstable.

### Production of 3,4-DHBA from glucose by the help of QsuB and N-QsuB

*E*. *coli* does not degrade 3,4-DHBA, so QsuB and N-QsuB enzymes were tested for 3,4-DHBA production in *E*. *coli* MG1655Δ*aroE* cells. Inactivation of the shikimate dehydrogenase encoded by the *aroE* gene provided DHS accumulation in this strain. Isogenic strains which was different only in the 3’-part deletion of *qsuB* gene were created. The genes *qsuB* and *n-qsuB* were under the control of the IPTG-inducible promoter P_*lac*_. The obtained strains were tested for 3,4-DHBA production by fermentation with glucose as the carbon source (Table 2).

**Table 2:** Production of DHS and 3,4-DHBA from glucose (40 g/l) in test tubes.

N	MG1655Δ*aroE* strain	OD_540_	DHS, g/l	3,4-DHBA, g/l	Res. glucose, g/l	IPTG*, 1 mM	CoCl_2_, μM
1	‒	31 ± 3	3.7 ± 0.1	< 0.1	5 ± 1	‒	‒
2		27 ± 4	4 ± 1	< 0.1	10 ± 1	+	
3		29 ± 1	5.1 ± 0.1	< 0.1	4.0 ± 0.1	‒	10
4		29 ± 1	5.0 ± 0.1	< 0.1	5 ± 1	+	
5		27 ± 1	4.6 ± 0.1	< 0.1	9 ± 1	‒	100
6		27 ± 2	5.0 ± 0.1	< 0.1	8 ± 2	+	
7	P_*lac*_-*qsuB*	28 ± 3	2.2 ± 0.1	1.0 ± 0.1	10 ± 1	‒	‒
8		30 ± 1	~ 0.1	3.2 ± 0.3	8 ± 3	+	
9		29 ± 1	3.2 ± 0.1	1.6 ± 0.1	6 ± 1	‒	10
10		29 ± 1	~ 0.2	3.9 ± 0.1	7 ± 1	+	
11		27 ± 1	2.2 ± 0.1	1.9 ± 0.1	8 ± 1	‒	100
12		27 ± 1	~ 0.2	3.4 ± 0.1	10 ± 1	+	
13	P_*lac*_-*n-qsuB*	27 ± 1	3.4 ± 0.2	~ 0.1	10 ± 1	‒	‒
14		31 ± 1	2.0 ± 0.1	1.2 ± 0.1	8 ± 4	+	
15		29 ± 1	5.1 ± 0.1	< 0.1	6 ± 1	‒	10
16		29 ± 3	4.0 ± 0.1	1.4 ± 0.1	6 ± 1	+	
17		28 ± 2	4.7 ± 0.1	~ 0.1	7 ± 1	‒	100
18		28 ± 3	3.0 ± 0.1	1.2 ± 0.1	8 ± 1	+	

Data were obtained after 44 h of incubation. SD below 0.1 is not given.

* IPTG was added to parallel tubes before inoculation.

As seen from Table 2, the MG1655Δ*aroE* strain (lines 1 ‒ 6) began to produce 3,4-DHBA after the introduction of the P_*lac*_-*qsuB* (lines 7–12) or the P_*lac*_-*n-qsuB* (lines 13–18) genes. The accumulation of 3,4-DHBA was higher in the MG1655*ΔaroE* P_lac_-*qsuB* strain. The production of 3,4-DHBA was tested in the fermentation medium initially containing Mg^2+^ (Table 2, lines 1, 2, 7, 8, 13, 14) and also with supplementation of Co^2+^, 10 μM (lines 3, 4, 9, 10, 15, 16) and 100 μM (lines 5, 6, 11, 12, 17, 18). The addition of Co^2+^ (10 μM was more preferable) increased the accumulation of both DHS and 3,4-DHBA. An increase in DHS biosynthesis could be due to the enhancement of 3-deoxy-D-arabino-heptulosonate 7-phosphate (DAHP) synthase activity. DAHP synthases that direct the carbon flow into aromatic pathway are metal-dependent enzymes. In particular, cobalt is one of the preferable cofactors for *E*. *coli* DAHP synthase AroG [34]. The positive effect of Co^2+^ on 3,4-DHBA production was more pronounced in the MG1655*ΔaroE* P_*lac*_*-qsuB* strain (Table 2, lines 9–12). Thisstrain produced 3,4-DHBA with and without IPTG addition, although the production was higher in the latter case. The MG1655*ΔaroE* P_*lac*_-*n-qsuB* strain produced 3,4-DHBA only in the presence of IPTG, and its accumulation was three times lower than that in the MG1655*ΔaroE* P_*lac*_-*qsuB* strain.

## Discussion

Our investigations showed that QsuB and its truncated variant N-QsuB containing the only N-terminal domain had a DSD activity. Kinetic analysis of QsuB confirmed that the enzyme was activated by divalent cations and revealed a preference for Co^2+^. Notably, Co^2+^ is the main cofactor of QuiC1 [15], while Mg^2+^ is preferred for AsbF of *B*. *thuringiensis* [12]. The divalent metal concentrations used in tests *in vitro* were obviously higher than physiologically relevant ones. Nevertheless, *C*. *glutamicum* was able to grow at 2 mM Co^2+^ and was more resistant to cobalt than *E*. *coli* (less 1 mM) [35]. Thus, cobalt could be the physiologically relevant metal cofactor for DSD of *C*. *glutamicum*.

The enzymatic activity of QsuB C-terminal domain was not manifested in 3,4-DHBA degradation tests *in vitro*. Removal of this domain led to changes in catalytic properties of DSD and in its product inhibition type. We associated these changes with the observed differences in the oligomeric structure of the full-length and the truncated enzymes. Analysis of QsuB and N-QsuB dimers modeled on the basis of QuiC1 dimer showed that the possibility of N-QsuB dimer formation was greatly reduced. QsuB dimer was assembled mainly due to formation of hydrogen bonds between the residues of N and C-terminal domains of two subunits. We also tested the specific activities of QsuB protein fractions corresponding to monomer, tetramer and octamer collected after SEC (35±3, 36±4, 46±7 μmol*min^-1^mg^-1^, respectively). QsuB oligomerization did not result in the increase of enzyme activity by itself. Despite the activity of N-QsuB was slightly higher than that of the QsuB enzyme at physiological pH (Fig 5B), the C-terminal domain was appeared to be sufficient for DSD activity in *E*. *coli* cells (Table 2). Therefore, it was proposed that C-terminal domain is necessary for protein oligomerization which maintains stable structure of the enzyme.

We determined that the QsuB enzyme could be inhibited by both the substrate and the product, but it could happen at mM range of intracellular concentrations. The inhibition constants for other DSDs were not reported, but they are important when choosing an enzyme for metabolic engineering. The QsuB enzyme relates to catabolic DSDs which possess higher enzymatic activity and lower affinity to DHS in comparison with biosynthetic DSD AsbF (Table 1 and [12, 14]). The low affinity of the QsuB enzyme to DHS did not affect the accumulation of 3,4-DHBA in *E*. *coli* Δ*aroE* cells where there was no competition for the substrate. In general, the enzymes with high activities are more preferable for microbial production.

## Supporting information

S1 Raw images Raw images used to generate Fig 3. (PDF)

S1 Table Primers used in the investigation. (PDF)

S1 File Verification of DSD reaction product. (PDF)

S2 File QsuB dimer formation analysis. (PDF)

S3 File N-QsuB dimer formation analysis. (PDF)
