# Supplementary material for: Characterization of the Corynebacterium glutamicum dehydroshikimate dehydratase QsuB and its potential for microbial production of protocatechuic acid
Source: PLoS One. 2020 Aug 21;15(8):e0231560. doi: 10.1371/journal.pone.0231560 (PMC7442255; doi:10.1371/journal.pone.0231560)
Supplement: S1 Raw images — (PDF) [file pone.0231560.s001.pdf]

Raw images used to generate Fig 3.

S1A Fig. SDS-PAGE gel of QsuB in crude extract. Lane M (marker) and lane 1 (QsuB) were used for Fig 3A preparation.

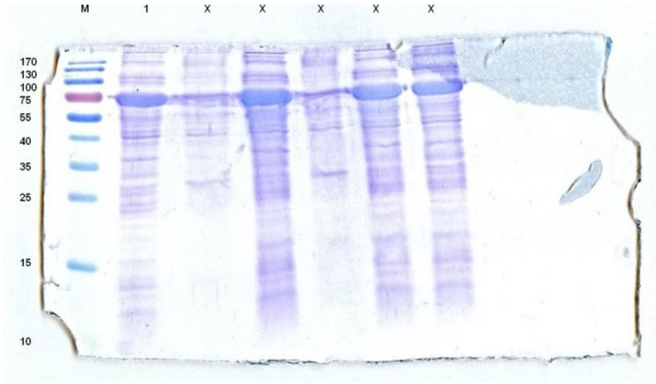

S1B Fig. SDS-PAGE gel of N-QsuB in crude extract. Lane M (marker) and lane 1 (N-QsuB) were used for Fig 3B preparation.

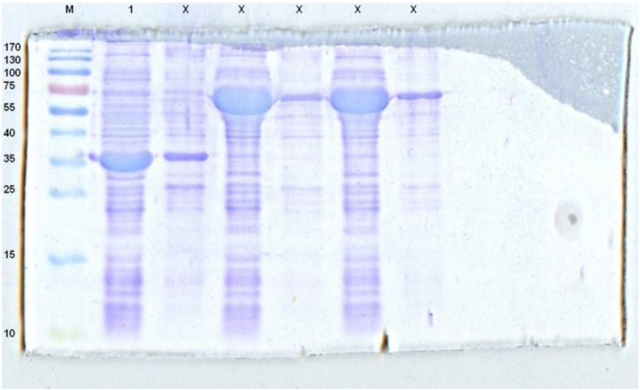

S1C Fig. SDS-PAGE gel of purified QsuB and N-QsuB. Lane M (marker), lane 1 (QsuB) and lane 2 (N-QsuB) were used for Fig 3C preparation.

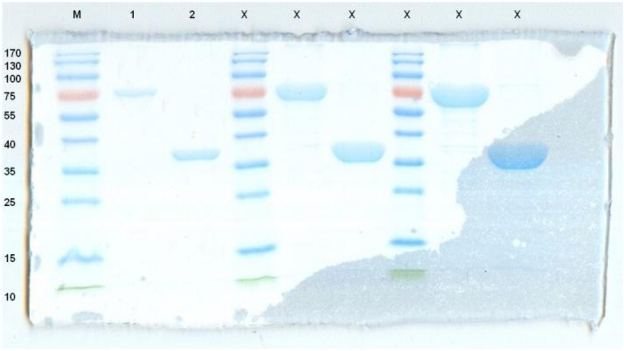

Raw images were obtained using photo scanner HP Scanjet G3010. Lanes X were excluded from the final image.
