# Supplementary material for: Characterization of the Corynebacterium glutamicum dehydroshikimate dehydratase QsuB and its potential for microbial production of protocatechuic acid
Source: PLoS One. 2020 Aug 21;15(8):e0231560. doi: 10.1371/journal.pone.0231560 (PMC7442255; doi:10.1371/journal.pone.0231560)
Supplement: S1 Table — (PDF) [file pone.0231560.s002.pdf]

S2 Table. Primers used in the investigation.

Primer	Sequence (5'–to 3'), restriction sites are underlined
P1	CATATGTATATCTCCTTCTTAAAGTTAAACAAAA
P2	CACCACCACCACCACCACTGAGATCCGGCTGCTAACAAAG
P3	TTTTGTTTAACTTTAAGAAGGAGATATACATATGCGTACATCCATTGCCACTGT
P4	GTGGTGGTGGTGGTGGTGGTTTGGGATTCCCCGCTCG
P5	GTGGTGGTGGTGGTGGTGGAAATCAACACCCTCAGGTTCC
P6	TTTTTGTCGACTCTAGAGGATCTGCGGGCAG
P7	CGCATATGTATATCTCCTTCTTAAATCTAGATCCTGTGTGAAATTGTTATCC
P8	TTTAAGAAGGAGATATACATATGCGTACATC
P9	TTTTTGAGCTCCTAGTTTGGGATTCCCCGCTCG
P10	GTTATAAAGCAATTGCAGGAGGAATTGTCCGCGTGACGCTCAAGTTAGTATAAAAAAGCTGAA
P11	GGCTATCGGATTACCAAAAACAGCATAGGTTTCCATTGAAGCCTGCTTTTTTATACTAAGTTG
P12	CTTCCTGAGGTCGCGGAACCTGAGGGTGTTGATTTCTAGCGCTCAAGTTAGTATAAAAAAGCTGAACGAGAAACG
P13	GTGATAAGCTGTCAAACATGAGAATTCGAGCTCCTATGAAGCCTGCTTTTTTATACTAAGTTGGCATTATAA
