# Supplementary material for: Characterization of the Corynebacterium glutamicum dehydroshikimate dehydratase QsuB and its potential for microbial production of protocatechuic acid
Source: PLoS One. 2020 Aug 21;15(8):e0231560. doi: 10.1371/journal.pone.0231560 (PMC7442255; doi:10.1371/journal.pone.0231560)

Verification of DSD reaction product

S3A Fig. UV-traces of DHS, 3,4-DHBA and DSD reaction products.

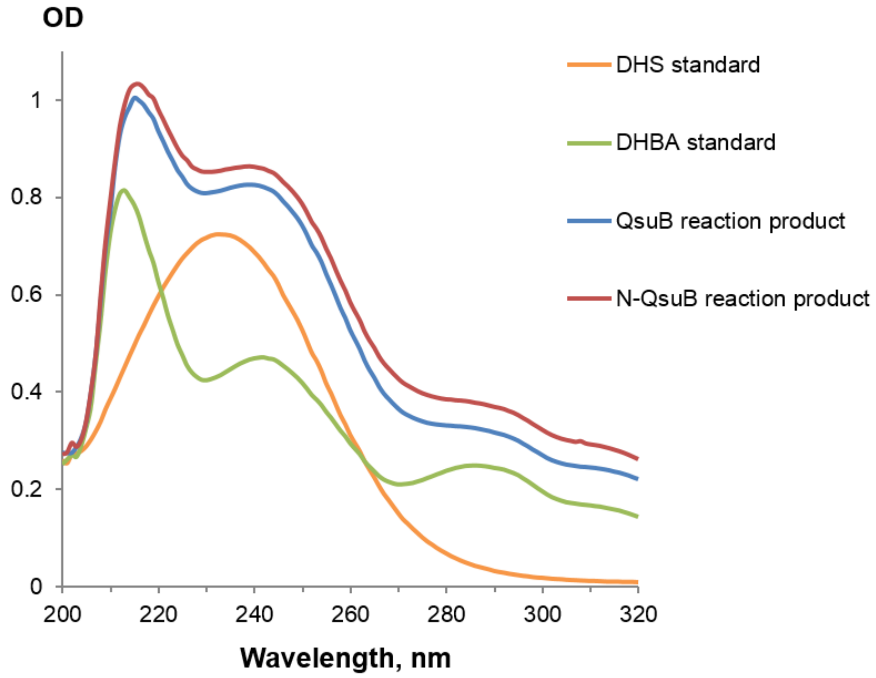

S3B Fig. HPLC elution times for DHS, 3,4-DHBA and DSD reaction products.

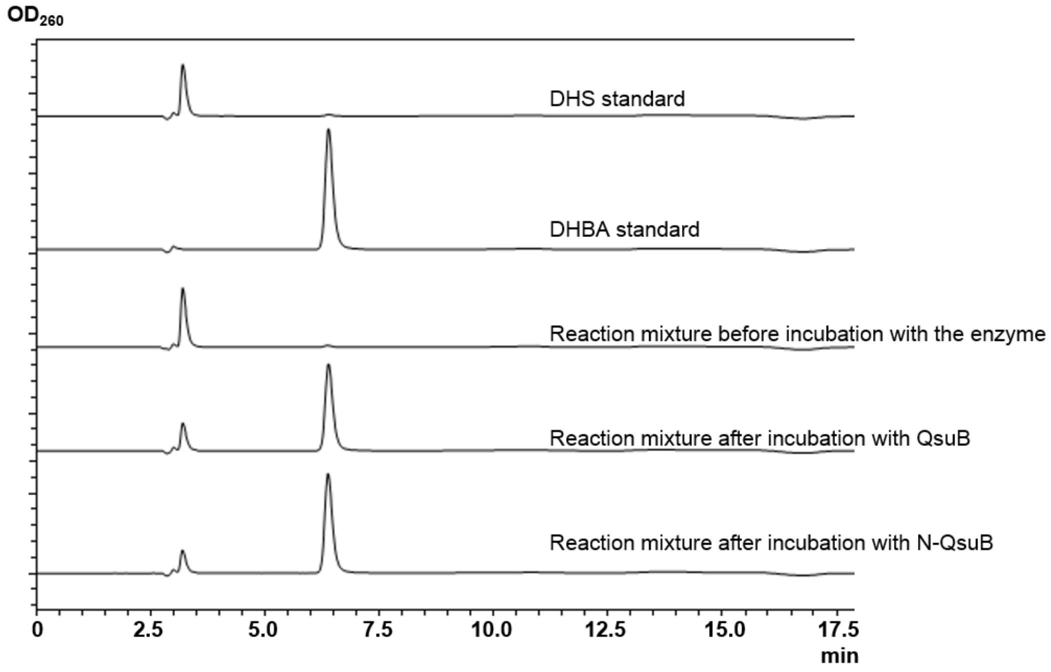

Supplement: S1 File — (PDF) [file pone.0231560.s003.pdf]
