# Supplementary material for: Characterization of the Corynebacterium glutamicum dehydroshikimate dehydratase QsuB and its potential for microbial production of protocatechuic acid
Source: PLoS One. 2020 Aug 21;15(8):e0231560. doi: 10.1371/journal.pone.0231560 (PMC7442255; doi:10.1371/journal.pone.0231560)
Supplement: S2 File — (PDF) [file pone.0231560.s004.pdf]

Protein Data Bank in Europe

PDBePISA

pdbe.org/pisa

Bringing Structure to Biology

PISA Interface.

interface # 1 in qsub_dim.pdb

interface #1/1

XML << < > >>

Interface Summary

XML

	Structure 1		Structure 2	
**Selection range**	A		B	
**class**	Protein		Protein	
**symmetry operation**	x,y,z		,,	
**symmetry ID**	1_555		0_555	
**Number of atoms**				
**interface**	282	5.7%	280	5.7%
**surface**	2867	58.3%	2884	58.7%
**total**	4917	100.0%	4917	100.0%
**Number of residues**				
**interface**	78	12.6%	78	12.6%
**surface**	584	94.5%	581	94.0%
**total**	618	100.0%	618	100.0%
**Solvent-accessible area, Å**				
**interface**	2528.9	9.4%	2542.3	9.4%
**total**	26980.1	100.0%	26972.5	100.0%
**Solvation energy, kcal/mol**				
**isolated structure**	-503.0	100.0%	-502.8	100.0%
**gain on complex formation**	-15.8	3.1%	-16.0	3.2%
**average gain**	-11.3	2.2%	-11.2	2.2%
**P-value**	0.144		0.124	

View structure 1 | interface | structure 2

Download structure 1 | interface | structure 2

This interface scored

0.000

in Complex Formation Significance Score (CSS).

CSS ranges from 0 to 1 as interface relevance to complex formation increases.

Achieved CSS implies that the interface does not play any role in complex formation and seems to be a resul

Hydrogen bonds

XML

##	Structure 1	Dist. [Å]	Structure 2
1	A:GLN 376[NE2]	3.22	B:VAL 38[O]
2	A:ARG 252[NH2]	2.02	B:ARG 246[O]
3	A:GLN 440[NE2]	2.40	B:ARG 246[O]
4	A:LYS 247[HZ3]	2.05	B:GLU 249[OE2]
5	A:ARG 246[H]	1.90	B:ASP 396[OD1]
6	A:PRO 466[N]	3.78	B:ASN 618[OD1]
7	A:VAL 38[O]	3.12	B:GLN 376[NE2]
8	A:ARG 246[O]	2.21	B:ARG 252[NH2]
9	A:ARG 246[O]	2.31	B:GLN 440[NE2]
10	A:GLU 249[OE2]	1.83	B:LYS 247[HZ3]
11	A:ASP 396[OD1]	1.96	B:ARG 246[H]
12	A:ASN 618[OD1]	3.63	B:PRO 466[N]

Salt bridges

XML

##	Structure 1	Dist. [Å]	Structure 2
1	A:LYS 247[NZ]	3.87	B:GLU 249[OE1]
2	A:LYS 247[NZ]	2.89	B:GLU 249[OE2]
3	A:GLU 249[OE1]	3.73	B:LYS 247[NZ]
4	A:GLU 249[OE2]	2.67	B:LYS 247[NZ]

No disulfide bonds found

No covalent bonds found

Interfacing residues (not a contact table)

XML Display level: Residues

Inaccessible residues — HSDC Residues making **H**ydrogen/**D**isulphide bond, **S**alt bridge or **C**ovalent link

Solvent-accessible residues — Interfacing residues

ASA Accessible Surface Area, Å² **BSA** Buried Surface Area, Å² **Δ^{i}G** Solvation energy effect, kcal/mol |||| Buried area percentage, one bar per 10%

##	Structure 1	HSDC	ASA	BSA	Δ^{i}G	##	Structure 2	HSDC	ASA	BSA	Δ^{i}G
1	A:MET 1		62.85	0.00	0.00	1	B:MET 1		63.44	0.00	0.00
2	A:ARG 2		75.81	0.00	0.00	2	B:ARG 2		75.40	0.00	0.00
3	A:THR 3		3.85	0.00	0.00	3	B:THR 3		4.69	0.00	0.00
4	A:SER 4		0.00	0.00	0.00	4	B:SER 4		0.00	0.00	0.00
5	A:ILE 5		1.34	0.00	0.00	5	B:ILE 5		1.17	0.00	0.00
6	A:ALA 6		0.00	0.00	0.00	6	B:ALA 6		0.00	0.00	0.00
7	A:THR 7		3.18	0.00	0.00	7	B:THR 7		3.18	0.00	0.00
8	A:VAL 8		17.42	0.00	0.00	8	B:VAL 8		17.69	0.00	0.00
9	A:CYS 9		0.00	0.00	0.00	9	B:CYS 9		0.00	0.00	0.00
10	A:LEU 10		4.65	0.00	0.00	10	B:LEU 10		5.01	0.00	0.00
11	A:SER 11		13.03	0.00	0.00	11	B:SER 11		13.02	0.00	0.00
12	A:GLY 12		21.90	0.00	0.00	12	B:GLY 12		22.09	0.00	0.00

<table>
<tr><td>13</td><td>A:THR</td><td>13</td><td></td><td>84.55</td><td>11.38 ||</td><td>0.18</td><td>13</td><td>B:THR</td><td>13</td><td></td><td>84.96</td><td>10.71 ||</td><td>0.17</td></tr>
<tr><td>14</td><td>A:LEU</td><td>14</td><td></td><td>7.64</td><td>0.00</td><td>0.00</td><td>14</td><td>B:LEU</td><td>14</td><td></td><td>7.31</td><td>0.00</td><td>0.00</td></tr>
<tr><td>15</td><td>A:ALA</td><td>15</td><td></td><td>48.47</td><td>0.00</td><td>0.00</td><td>15</td><td>B:ALA</td><td>15</td><td></td><td>50.00</td><td>0.00</td><td>0.00</td></tr>
<tr><td>16</td><td>A:GLU</td><td>16</td><td></td><td>55.79</td><td>16.79 ||||</td><td>-0.01</td><td>16</td><td>B:GLU</td><td>16</td><td></td><td>56.07</td><td>16.85 ||||</td><td>-0.01</td></tr>
<tr><td>17</td><td>A:LYS</td><td>17</td><td></td><td>10.86</td><td>0.00</td><td>0.00</td><td>17</td><td>B:LYS</td><td>17</td><td></td><td>10.85</td><td>0.00</td><td>0.00</td></tr>
<tr><td>18</td><td>A:LEU</td><td>18</td><td></td><td>2.85</td><td>0.00</td><td>0.00</td><td>18</td><td>B:LEU</td><td>18</td><td></td><td>2.68</td><td>0.00</td><td>0.00</td></tr>
<tr><td>19</td><td>A:ARG</td><td>19</td><td></td><td>136.29</td><td>21.86 ||</td><td>-0.10</td><td>19</td><td>B:ARG</td><td>19</td><td></td><td>137.33</td><td>22.53 ||</td><td>-0.07</td></tr>
<tr><td>20</td><td>A:ALA</td><td>20</td><td></td><td>8.95</td><td>0.00</td><td>0.00</td><td>20</td><td>B:ALA</td><td>20</td><td></td><td>9.28</td><td>0.00</td><td>0.00</td></tr>
<tr><td>21</td><td>A:ALA</td><td>21</td><td></td><td>1.34</td><td>0.00</td><td>0.00</td><td>21</td><td>B:ALA</td><td>21</td><td></td><td>1.17</td><td>0.00</td><td>0.00</td></tr>
<tr><td>22</td><td>A:ALA</td><td>22</td><td></td><td>23.77</td><td>0.00</td><td>0.00</td><td>22</td><td>B:ALA</td><td>22</td><td></td><td>22.53</td><td>0.00</td><td>0.00</td></tr>
<tr><td>23</td><td>A:ASP</td><td>23</td><td></td><td>65.92</td><td>0.00</td><td>0.00</td><td>23</td><td>B:ASP</td><td>23</td><td></td><td>65.53</td><td>0.00</td><td>0.00</td></tr>
<tr><td>24</td><td>A:ALA</td><td>24</td><td></td><td>1.76</td><td>0.00</td><td>0.00</td><td>24</td><td>B:ALA</td><td>24</td><td></td><td>1.89</td><td>0.00</td><td>0.00</td></tr>
<tr><td>25</td><td>A:GLY</td><td>25</td><td></td><td>45.18</td><td>0.00</td><td>0.00</td><td>25</td><td>B:GLY</td><td>25</td><td></td><td>44.95</td><td>0.00</td><td>0.00</td></tr>
<tr><td>26</td><td>A:PHE</td><td>26</td><td></td><td>10.64</td><td>0.00</td><td>0.00</td><td>26</td><td>B:PHE</td><td>26</td><td></td><td>10.81</td><td>0.00</td><td>0.00</td></tr>
<tr><td>27</td><td>A:ASP</td><td>27</td><td></td><td>59.04</td><td>0.00</td><td>0.00</td><td>27</td><td>B:ASP</td><td>27</td><td></td><td>59.19</td><td>0.00</td><td>0.00</td></tr>
<tr><td>28</td><td>A:GLY</td><td>28</td><td></td><td>0.00</td><td>0.00</td><td>0.00</td><td>28</td><td>B:GLY</td><td>28</td><td></td><td>0.00</td><td>0.00</td><td>0.00</td></tr>
<tr><td>29</td><td>A:VAL</td><td>29</td><td></td><td>1.84</td><td>0.00</td><td>0.00</td><td>29</td><td>B:VAL</td><td>29</td><td></td><td>2.18</td><td>0.00</td><td>0.00</td></tr>
<tr><td>30</td><td>A:GLU</td><td>30</td><td></td><td>0.00</td><td>0.00</td><td>0.00</td><td>30</td><td>B:GLU</td><td>30</td><td></td><td>0.00</td><td>0.00</td><td>0.00</td></tr>
<tr><td>31</td><td>A:ILE</td><td>31</td><td></td><td>3.35</td><td>0.00</td><td>0.00</td><td>31</td><td>B:ILE</td><td>31</td><td></td><td>3.01</td><td>0.00</td><td>0.00</td></tr>
<tr><td>32</td><td>A:PHE</td><td>32</td><td></td><td>21.55</td><td>0.00</td><td>0.00</td><td>32</td><td>B:PHE</td><td>32</td><td></td><td>21.39</td><td>0.00</td><td>0.00</td></tr>
<tr><td>33</td><td>A:GLU</td><td>33</td><td></td><td>35.60</td><td>3.80 ||</td><td>-0.06</td><td>33</td><td>B:GLU</td><td>33</td><td></td><td>34.86</td><td>2.09 |</td><td>-0.04</td></tr>
<tr><td>34</td><td>A:GLN</td><td>34</td><td></td><td>109.80</td><td>19.01 ||</td><td>-0.32</td><td>34</td><td>B:GLN</td><td>34</td><td></td><td>110.34</td><td>17.06 ||</td><td>-0.29</td></tr>
<tr><td>35</td><td>A:ASP</td><td>35</td><td></td><td>10.51</td><td>0.00</td><td>0.00</td><td>35</td><td>B:ASP</td><td>35</td><td></td><td>11.02</td><td>0.00</td><td>0.00</td></tr>
<tr><td>36</td><td>A:LEU</td><td>36</td><td></td><td>23.92</td><td>3.31 ||</td><td>-0.04</td><td>36</td><td>B:LEU</td><td>36</td><td></td><td>24.90</td><td>3.31 ||</td><td>-0.04</td></tr>
<tr><td>37</td><td>A:VAL</td><td>37</td><td></td><td>120.34</td><td>61.75 ||||||</td><td>0.39</td><td>37</td><td>B:VAL</td><td>37</td><td></td><td>119.09</td><td>61.99 ||||||</td><td>0.40</td></tr>
<tr><td>38</td><td>A:VAL</td><td>38</td><td>H</td><td>101.06</td><td>68.63 |||||||</td><td>0.70</td><td>38</td><td>B:VAL</td><td>38</td><td>H</td><td>101.94</td><td>68.50 |||||||</td><td>0.71</td></tr>
<tr><td>39</td><td>A:SER</td><td>39</td><td></td><td>21.41</td><td>9.79 |||||</td><td>-0.05</td><td>39</td><td>B:SER</td><td>39</td><td></td><td>20.97</td><td>8.34 ||||</td><td>-0.05</td></tr>
<tr><td>40</td><td>A:PRO</td><td>40</td><td></td><td>135.65</td><td>40.47 |||</td><td>0.64</td><td>40</td><td>B:PRO</td><td>40</td><td></td><td>132.92</td><td>36.46 |||</td><td>0.58</td></tr>
<tr><td>41</td><td>A:HIS</td><td>41</td><td></td><td>87.73</td><td>0.00</td><td>0.00</td><td>41</td><td>B:HIS</td><td>41</td><td></td><td>89.22</td><td>0.00</td><td>0.00</td></tr>
<tr><td>42</td><td>A:SER</td><td>42</td><td></td><td>58.75</td><td>0.00</td><td>0.00</td><td>42</td><td>B:SER</td><td>42</td><td></td><td>57.43</td><td>0.00</td><td>0.00</td></tr>
<tr><td>43</td><td>A:ALA</td><td>43</td><td></td><td>18.59</td><td>0.00</td><td>0.00</td><td>43</td><td>B:ALA</td><td>43</td><td></td><td>18.40</td><td>0.00</td><td>0.00</td></tr>
<tr><td>44</td><td>A:GLU</td><td>44</td><td></td><td>54.91</td><td>0.00</td><td>0.00</td><td>44</td><td>B:GLU</td><td>44</td><td></td><td>56.52</td><td>0.00</td><td>0.00</td></tr>
<tr><td>45</td><td>A:GLN</td><td>45</td><td></td><td>81.41</td><td>0.00</td><td>0.00</td><td>45</td><td>B:GLN</td><td>45</td><td></td><td>81.38</td><td>0.00</td><td>0.00</td></tr>
<tr><td>46</td><td>A:ILE</td><td>46</td><td></td><td>3.67</td><td>0.00</td><td>0.00</td><td>46</td><td>B:ILE</td><td>46</td><td></td><td>4.33</td><td>0.00</td><td>0.00</td></tr>
<tr><td>47</td><td>A:ARG</td><td>47</td><td></td><td>63.39</td><td>0.00</td><td>0.00</td><td>47</td><td>B:ARG</td><td>47</td><td></td><td>61.34</td><td>0.00</td><td>0.00</td></tr>
<tr><td>48</td><td>A:GLN</td><td>48</td><td></td><td>81.90</td><td>0.00</td><td>0.00</td><td>48</td><td>B:GLN</td><td>48</td><td></td><td>82.55</td><td>0.00</td><td>0.00</td></tr>
<tr><td>49</td><td>A:ARG</td><td>49</td><td></td><td>72.01</td><td>0.00</td><td>0.00</td><td>49</td><td>B:ARG</td><td>49</td><td></td><td>73.17</td><td>0.00</td><td>0.00</td></tr>
<tr><td>50</td><td>A:ALA</td><td>50</td><td></td><td>1.50</td><td>0.00</td><td>0.00</td><td>50</td><td>B:ALA</td><td>50</td><td></td><td>1.66</td><td>0.00</td><td>0.00</td></tr>
<tr><td>51</td><td>A:GLN</td><td>51</td><td></td><td>104.25</td><td>0.00</td><td>0.00</td><td>51</td><td>B:GLN</td><td>51</td><td></td><td>106.95</td><td>0.00</td><td>0.00</td></tr>
<tr><td>52</td><td>A:ASP</td><td>52</td><td></td><td>117.23</td><td>0.00</td><td>0.00</td><td>52</td><td>B:ASP</td><td>52</td><td></td><td>117.38</td><td>0.00</td><td>0.00</td></tr>
<tr><td>53</td><td>A:LEU</td><td>53</td><td></td><td>59.37</td><td>0.00</td><td>0.00</td><td>53</td><td>B:LEU</td><td>53</td><td></td><td>59.77</td><td>0.00</td><td>0.00</td></tr>
<tr><td>54</td><td>A:GLY</td><td>54</td><td></td><td>55.37</td><td>0.00</td><td>0.00</td><td>54</td><td>B:GLY</td><td>54</td><td></td><td>56.29</td><td>0.00</td><td>0.00</td></tr>
<tr><td>55</td><td>A:LEU</td><td>55</td><td></td><td>3.43</td><td>0.00</td><td>0.00</td><td>55</td><td>B:LEU</td><td>55</td><td></td><td>3.48</td><td>0.00</td><td>0.00</td></tr>
<tr><td>56</td><td>A:THR</td><td>56</td><td></td><td>45.67</td><td>0.00</td><td>0.00</td><td>56</td><td>B:THR</td><td>56</td><td></td><td>45.95</td><td>0.00</td><td>0.00</td></tr>
<tr><td>57</td><td>A:LEU</td><td>57</td><td></td><td>6.15</td><td>0.00</td><td>0.00</td><td>57</td><td>B:LEU</td><td>57</td><td></td><td>5.54</td><td>0.00</td><td>0.00</td></tr>
<tr><td>58</td><td>A:ASP</td><td>58</td><td></td><td>10.71</td><td>0.00</td><td>0.00</td><td>58</td><td>B:ASP</td><td>58</td><td></td><td>10.73</td><td>0.00</td><td>0.00</td></tr>
<tr><td>59</td><td>A:LEU</td><td>59</td><td></td><td>0.16</td><td>0.00</td><td>0.00</td><td>59</td><td>B:LEU</td><td>59</td><td></td><td>0.16</td><td>0.00</td><td>0.00</td></tr>
<tr><td>60</td><td>A:PHE</td><td>60</td><td></td><td>0.16</td><td>0.00</td><td>0.00</td><td>60</td><td>B:PHE</td><td>60</td><td></td><td>0.16</td><td>0.00</td><td>0.00</td></tr>
<tr><td>61</td><td>A:GLN</td><td>61</td><td></td><td>5.04</td><td>0.00</td><td>0.00</td><td>61</td><td>B:GLN</td><td>61</td><td></td><td>4.36</td><td>0.00</td><td>0.00</td></tr>
<tr><td>62</td><td>A:PRO</td><td>62</td><td></td><td>29.58</td><td>11.52 ||||</td><td>-0.06</td><td>62</td><td>B:PRO</td><td>62</td><td></td><td>28.58</td><td>10.95 ||||</td><td>-0.05</td></tr>
<tr><td>63</td><td>A:PHE</td><td>63</td><td></td><td>2.25</td><td>0.00</td><td>0.00</td><td>63</td><td>B:PHE</td><td>63</td><td></td><td>2.39</td><td>0.00</td><td>0.00</td></tr>
<tr><td>64</td><td>A:ARG</td><td>64</td><td></td><td>70.16</td><td>10.31 ||</td><td>0.16</td><td>64</td><td>B:ARG</td><td>64</td><td></td><td>68.45</td><td>11.63 ||</td><td>0.17</td></tr>
<tr><td>65</td><td>A:ASP</td><td>65</td><td></td><td>43.31</td><td>1.23 |</td><td>-0.02</td><td>65</td><td>B:ASP</td><td>65</td><td></td><td>43.04</td><td>2.21 |</td><td>-0.04</td></tr>
<tr><td>66</td><td>A:PHE</td><td>66</td><td></td><td>1.47</td><td>0.00</td><td>0.00</td><td>66</td><td>B:PHE</td><td>66</td><td></td><td>1.26</td><td>0.00</td><td>0.00</td></tr>
<tr><td>67</td><td>A:GLU</td><td>67</td><td></td><td>0.00</td><td>0.00</td><td>0.00</td><td>67</td><td>B:GLU</td><td>67</td><td></td><td>0.00</td><td>0.00</td><td>0.00</td></tr>
<tr><td>68</td><td>A:GLY</td><td>68</td><td></td><td>4.13</td><td>0.00</td><td>0.00</td><td>68</td><td>B:GLY</td><td>68</td><td></td><td>3.89</td><td>0.00</td><td>0.00</td></tr>
<tr><td>69</td><td>A:VAL</td><td>69</td><td></td><td>30.90</td><td>0.00</td><td>0.00</td><td>69</td><td>B:VAL</td><td>69</td><td></td><td>31.70</td><td>0.00</td><td>0.00</td></tr>
<tr><td>70</td><td>A:GLU</td><td>70</td><td></td><td>99.53</td><td>0.00</td><td>0.00</td><td>70</td><td>B:GLU</td><td>70</td><td></td><td>102.44</td><td>0.00</td><td>0.00</td></tr>
<tr><td>71</td><td>A:GLU</td><td>71</td><td></td><td>91.54</td><td>0.00</td><td>0.00</td><td>71</td><td>B:GLU</td><td>71</td><td></td><td>91.81</td><td>0.00</td><td>0.00</td></tr>
<tr><td>72</td><td>A:GLU</td><td>72</td><td></td><td>113.15</td><td>0.00</td><td>0.00</td><td>72</td><td>B:GLU</td><td>72</td><td></td><td>113.55</td><td>0.00</td><td>0.00</td></tr>
<tr><td>73</td><td>A:GLN</td><td>73</td><td></td><td>57.93</td><td>0.00</td><td>0.00</td><td>73</td><td>B:GLN</td><td>73</td><td></td><td>58.78</td><td>0.00</td><td>0.00</td></tr>
<tr><td>74</td><td>A:PHE</td><td>74</td><td></td><td>31.46</td><td>0.00</td><td>0.00</td><td>74</td><td>B:PHE</td><td>74</td><td></td><td>31.63</td><td>0.00</td><td>0.00</td></tr>
<tr><td>75</td><td>A:LEU</td><td>75</td><td></td><td>127.98</td><td>0.00</td><td>0.00</td><td>75</td><td>B:LEU</td><td>75</td><td></td><td>128.29</td><td>0.00</td><td>0.00</td></tr>
<tr><td>76</td><td>A:LYS</td><td>76</td><td></td><td>106.14</td><td>0.00</td><td>0.00</td><td>76</td><td>B:LYS</td><td>76</td><td></td><td>105.69</td><td>0.00</td><td>0.00</td></tr>
<tr><td>77</td><td>A:ASN</td><td>77</td><td></td><td>12.67</td><td>0.00</td><td>0.00</td><td>77</td><td>B:ASN</td><td>77</td><td></td><td>12.65</td><td>0.00</td><td>0.00</td></tr>
<tr><td>78</td><td>A:LEU</td><td>78</td><td></td><td>34.63</td><td>0.00</td><td>0.00</td><td>78</td><td>B:LEU</td><td>78</td><td></td><td>35.12</td><td>0.00</td><td>0.00</td></tr>
<tr><td>79</td><td>A:HIS</td><td>79</td><td></td><td>102.41</td><td>0.00</td><td>0.00</td><td>79</td><td>B:HIS</td><td>79</td><td></td><td>102.46</td><td>0.00</td><td>0.00</td></tr>
<tr><td>80</td><td>A:ARG</td><td>80</td><td></td><td>90.52</td><td>24.50 |||</td><td>-0.58</td><td>80</td><td>B:ARG</td><td>80</td><td></td><td>91.08</td><td>23.79 |||</td><td>-0.58</td></tr>
<tr><td>81</td><td>A:LEU</td><td>81</td><td></td><td>0.00</td><td>0.00</td><td>0.00</td><td>81</td><td>B:LEU</td><td>81</td><td></td><td>0.00</td><td>0.00</td><td>0.00</td></tr>
<tr><td>82</td><td>A:GLU</td><td>82</td><td></td><td>31.73</td><td>0.00</td><td>0.00</td><td>82</td><td>B:GLU</td><td>82</td><td></td><td>31.77</td><td>0.00</td><td>0.00</td></tr>
<tr><td>83</td><td>A:GLU</td><td>83</td><td></td><td>79.96</td><td>0.00</td><td>0.00</td><td>83</td><td>B:GLU</td><td>83</td><td></td><td>81.42</td><td>0.00</td><td>0.00</td></tr>
<tr><td>84</td><td>A:LYS</td><td>84</td><td></td><td>25.82</td><td>0.00</td><td>0.00</td><td>84</td><td>B:LYS</td><td>84</td><td></td><td>25.68</td><td>0.00</td><td>0.00</td></tr>
<tr><td>85</td><td>A:PHE</td><td>85</td><td></td><td>1.60</td><td>0.00</td><td>0.00</td><td>85</td><td>B:PHE</td><td>85</td><td></td><td>2.33</td><td>0.00</td><td>0.00</td></tr>
<tr><td>86</td><td>A:LYS</td><td>86</td><td></td><td>84.28</td><td>0.00</td><td>0.00</td><td>86</td><td>B:LYS</td><td>86</td><td></td><td>84.04</td><td>0.00</td><td>0.00</td></tr>
<tr><td>87</td><td>A:LEU</td><td>87</td><td></td><td>53.27</td><td>0.00</td><td>0.00</td><td>87</td><td>B:LEU</td><td>87</td><td></td><td>53.50</td><td>0.00</td><td>0.00</td></tr>
<tr><td>88</td><td>A:MET</td><td>88</td><td></td><td>0.00</td><td>0.00</td><td>0.00</td><td>88</td><td>B:MET</td><td>88</td><td></td><td>0.00</td><td>0.00</td><td>0.00</td></tr>
<tr><td>89</td><td>A:ASN</td><td>89</td><td></td><td>95.73</td><td>0.00</td><td>0.00</td><td>89</td><td>B:ASN</td><td>89</td><td></td><td>95.60</td><td>0.00</td><td>0.00</td></tr>
<tr><td>90</td><td>A:ARG</td><td>90</td><td></td><td>115.79</td><td>0.00</td><td>0.00</td><td>90</td><td>B:ARG</td><td>90</td><td></td><td>115.69</td><td>0.00</td><td>0.00</td></tr>
<tr><td>91</td><td>A:LEU</td><td>91</td><td></td><td>0.12</td><td>0.00</td><td>0.00</td><td>91</td><td>B:LEU</td><td>91</td><td></td><td>0.12</td><td>0.00</td><td>0.00</td></tr>
<tr><td>92</td><td>A:GLY</td><td>92</td><td></td><td>41.04</td><td>0.00</td><td>0.00</td><td>92</td><td>B:GLY</td><td>92</td><td></td><td>41.04</td><td>0.00</td><td>0.00</td></tr>
<tr><td>93</td><td>A:ILE</td><td>93</td><td></td><td>22.58</td><td>0.00</td><td>0.00</td><td>93</td><td>B:ILE</td><td>93</td><td></td><td>22.54</td><td>0.00</td><td>0.00</td></tr>
</table>

94	A:GLU 94	76.00	0.00	0.00	94	B:GLU 94	74.63	0.00	0.00
95	A:MET 95	30.31	0.00	0.00	95	B:MET 95	30.14	0.00	0.00
96	A:ILE 96	0.17	0.00	0.00	96	B:ILE 96	0.17	0.00	0.00
97	A:LEU 97	2.51	0.00	0.00	97	B:LEU 97	2.35	0.00	0.00
98	A:LEU 98	0.00	0.00	0.00	98	B:LEU 98	0.00	0.00	0.00
99	A:CYS 99	0.00	0.00	0.00	99	B:CYS 99	0.00	0.00	0.00
100	A:SER 100	0.12	0.00	0.00	100	B:SER 100	0.12	0.00	0.00
101	A:ASN 101	0.91	0.00	0.00	101	B:ASN 101	1.47	0.00	0.00
102	A:VAL 102	52.71	0.00	0.00	102	B:VAL 102	54.70	0.00	0.00
103	A:GLY 103	30.86	0.00	0.00	103	B:GLY 103	31.18	0.00	0.00
104	A:THR 104	121.63	0.00	0.00	104	B:THR 104	120.85	0.00	0.00
105	A:ALA 105	88.32	0.00	0.00	105	B:ALA 105	88.42	0.00	0.00
106	A:THR 106	10.62	0.00	0.00	106	B:THR 106	9.94	0.00	0.00
107	A:ILE 107	85.97	0.00	0.00	107	B:ILE 107	85.53	0.00	0.00
108	A:ASN 108	83.36	0.00	0.00	108	B:ASN 108	81.78	0.00	0.00
109	A:ASP 109	64.79	0.00	0.00	109	B:ASP 109	65.18	0.00	0.00
110	A:ASP 110	63.15	0.00	0.00	110	B:ASP 110	62.86	0.00	0.00
111	A:ASP 111	87.41	0.00	0.00	111	B:ASP 111	87.36	0.00	0.00
112	A:LEU 112	92.47	0.00	0.00	112	B:LEU 112	93.11	0.00	0.00
113	A:PHE 113	0.62	0.00	0.00	113	B:PHE 113	0.47	0.00	0.00
114	A:VAL 114	14.89	0.00	0.00	114	B:VAL 114	15.00	0.00	0.00
115	A:GLU 115	72.62	0.00	0.00	115	B:GLU 115	71.36	0.00	0.00
116	A:GLN 116	9.45	0.00	0.00	116	B:GLN 116	9.20	0.00	0.00
117	A:LEU 117	0.00	0.00	0.00	117	B:LEU 117	0.00	0.00	0.00
118	A:HIS 118	68.31	0.00	0.00	118	B:HIS 118	67.51	0.00	0.00
119	A:ARG 119	65.66	0.00	0.00	119	B:ARG 119	65.33	0.00	0.00
120	A:ALA 120	0.00	0.00	0.00	120	B:ALA 120	0.00	0.00	0.00
121	A:ALA 121	0.00	0.00	0.00	121	B:ALA 121	0.00	0.00	0.00
122	A:ASP 122	46.60	0.00	0.00	122	B:ASP 122	47.14	0.00	0.00
123	A:LEU 123	46.95	0.00	0.00	123	B:LEU 123	47.09	0.00	0.00
124	A:ALA 124	0.00	0.00	0.00	124	B:ALA 124	0.00	0.00	0.00
125	A:GLU 125	67.19	0.00	0.00	125	B:GLU 125	65.78	0.00	0.00
126	A:LYS 126	137.08	0.00	0.00	126	B:LYS 126	136.59	0.00	0.00
127	A:TYR 127	91.12	0.00	0.00	127	B:TYR 127	90.84	0.00	0.00
128	A:ASN 128	102.69	0.00	0.00	128	B:ASN 128	103.19	0.00	0.00
129	A:VAL 129	7.83	0.00	0.00	129	B:VAL 129	8.38	0.00	0.00
130	A:LYS 130	36.84	0.00	0.00	130	B:LYS 130	36.63	0.00	0.00
131	A:ILE 131	0.00	0.00	0.00	131	B:ILE 131	0.00	0.00	0.00
132	A:ALA 132	0.17	0.00	0.00	132	B:ALA 132	0.00	0.00	0.00
133	A:TYR 133	0.00	0.00	0.00	133	B:TYR 133	0.00	0.00	0.00
134	A:GLU 134	9.39	0.00	0.00	134	B:GLU 134	9.82	0.00	0.00
135	A:ALA 135	2.30	0.00	0.00	135	B:ALA 135	1.38	0.00	0.00
136	A:LEU 136	1.50	0.00	0.00	136	B:LEU 136	1.68	0.00	0.00
137	A:ALA 137	8.96	0.00	0.00	137	B:ALA 137	9.33	0.00	0.00
138	A:TRP 138	79.20	0.00	0.00	138	B:TRP 138	81.40	0.00	0.00
139	A:GLY 139	5.26	0.00	0.00	139	B:GLY 139	5.47	0.00	0.00
140	A:LYS 140	75.94	0.00	0.00	140	B:LYS 140	76.18	0.00	0.00
141	A:PHE 141	64.21	0.00	0.00	141	B:PHE 141	64.43	0.00	0.00
142	A:VAL 142	1.17	0.00	0.00	142	B:VAL 142	0.84	0.00	0.00
143	A:ASN 143	45.81	0.00	0.00	143	B:ASN 143	45.30	0.00	0.00
144	A:ASP 144	11.84	0.00	0.00	144	B:ASP 144	11.29	0.00	0.00
145	A:PHE 145	3.12	0.00	0.00	145	B:PHE 145	3.09	0.00	0.00
146	A:GLU 146	48.75	0.00	0.00	146	B:GLU 146	48.34	0.00	0.00
147	A:HIS 147	69.64	0.00	0.00	147	B:HIS 147	69.81	0.00	0.00
148	A:ALA 148	0.00	0.00	0.00	148	B:ALA 148	0.00	0.00	0.00
149	A:HIS 149	33.97	0.00	0.00	149	B:HIS 149	33.04	0.00	0.00
150	A:ALA 150	58.01	0.00	0.00	150	B:ALA 150	58.77	0.00	0.00
151	A:LEU 151	7.04	0.00	0.00	151	B:LEU 151	6.70	0.00	0.00
152	A:VAL 152	3.82	0.00	0.00	152	B:VAL 152	3.48	0.00	0.00
153	A:GLU 153	55.99	0.00	0.00	153	B:GLU 153	57.44	0.00	0.00
154	A:LYS 154	91.33	0.00	0.00	154	B:LYS 154	90.37	0.00	0.00
155	A:VAL 155	4.33	0.00	0.00	155	B:VAL 155	4.04	0.00	0.00
156	A:ASN 156	123.79	0.00	0.00	156	B:ASN 156	124.12	0.00	0.00
157	A:HIS 157	27.39	0.00	0.00	157	B:HIS 157	27.20	0.00	0.00
158	A:LYS 158	145.89	0.00	0.00	158	B:LYS 158	146.11	0.00	0.00
159	A:ALA 159	1.67	0.00	0.00	159	B:ALA 159	1.67	0.00	0.00
160	A:LEU 160	2.82	0.00	0.00	160	B:LEU 160	3.17	0.00	0.00
161	A:GLY 161	0.00	0.00	0.00	161	B:GLY 161	0.00	0.00	0.00
162	A:THR 162	0.12	0.00	0.00	162	B:THR 162	0.12	0.00	0.00
163	A:CYS 163	0.98	0.00	0.00	163	B:CYS 163	1.64	0.00	0.00
164	A:LEU 164	2.35	0.00	0.00	164	B:LEU 164	2.18	0.00	0.00
165	A:ASP 165	8.87	0.00	0.00	165	B:ASP 165	8.94	0.00	0.00
166	A:THR 166	0.50	0.00	0.00	166	B:THR 166	0.33	0.00	0.00
167	A:PHE 167	2.00	0.00	0.00	167	B:PHE 167	1.84	0.00	0.00
168	A:HIS 168	12.05	0.00	0.00	168	B:HIS 168	11.70	0.00	0.00
169	A:ILE 169	5.19	0.00	0.00	169	B:ILE 169	5.53	0.00	0.00
170	A:LEU 170	21.72	0.00	0.00	170	B:LEU 170	21.02	0.00	0.00
171	A:SER 171	24.01	0.00	0.00	171	B:SER 171	24.24	0.00	0.00
172	A:ARG 172	73.04	0.00	0.00	172	B:ARG 172	74.67	0.00	0.00
173	A:GLY 173	57.76	0.00	0.00	173	B:GLY 173	58.03	0.00	0.00
174	A:TRP 174	96.01	0.00	0.00	174	B:TRP 174	98.46	0.00	0.00

175	A:GLU 175		87.46	0.00		0.00	175	B:GLU 175		87.95	0.00		0.00
176	A:THR 176		1.17	0.00		0.00	176	B:THR 176		1.34	0.00		0.00
177	A:ASP 177		52.01	0.00		0.00	177	B:ASP 177		52.86	0.00		0.00
178	A:GLU 178		85.23	0.00		0.00	178	B:GLU 178		85.38	0.00		0.00
179	A:VAL 179		0.67	0.00		0.00	179	B:VAL 179		0.84	0.00		0.00
180	A:GLU 180		72.56	0.00		0.00	180	B:GLU 180		72.17	0.00		0.00
181	A:ASN 181		110.72	0.00		0.00	181	B:ASN 181		111.15	0.00		0.00
182	A:ILE 182		9.99	0.00		0.00	182	B:ILE 182		9.73	0.00		0.00
183	A:PRO 183		47.30	0.00		0.00	183	B:PRO 183		47.13	0.00		0.00
184	A:ALA 184		22.91	0.00		0.00	184	B:ALA 184		22.60	0.00		0.00
185	A:GLU 185		118.86	0.00		0.00	185	B:GLU 185		117.94	0.00		0.00
186	A:LYS 186		48.02	0.00		0.00	186	B:LYS 186		47.52	0.00		0.00
187	A:ILE 187		0.00	0.00		0.00	187	B:ILE 187		0.00	0.00		0.00
188	A:PHE 188		28.10	0.00		0.00	188	B:PHE 188		27.14	0.00		0.00
189	A:PHE 189		1.10	0.00		0.00	189	B:PHE 189		1.26	0.00		0.00
190	A:VAL 190		1.00	0.00		0.00	190	B:VAL 190		1.17	0.00		0.00
191	A:GLN 191		3.87	0.00		0.00	191	B:GLN 191		3.63	0.00		0.00
192	A:LEU 192		0.00	0.00		0.00	192	B:LEU 192		0.00	0.00		0.00
193	A:ALA 193		0.00	0.00		0.00	193	B:ALA 193		0.00	0.00		0.00
194	A:ASP 194		3.27	0.00		0.00	194	B:ASP 194		3.81	0.00		0.00
195	A:ALA 195		0.00	0.00		0.00	195	B:ALA 195		0.00	0.00		0.00
196	A:PRO 196		21.05	0.00		0.00	196	B:PRO 196		21.05	0.00		0.00
197	A:LYS 197		91.65	0.00		0.00	197	B:LYS 197		93.34	0.00		0.00
198	A:LEU 198		87.91	30.02	||||	0.48	198	B:LEU 198		87.93	30.68	||||	0.49
199	A:SER 199		124.97	26.80	|||	0.13	199	B:SER 199		125.95	28.62	|||	0.15
200	A:MET 200		98.23	79.87	|||||||||	1.80	200	B:MET 200		98.90	79.81	|||||||||	1.80
201	A:ASP 201		85.70	0.98	|	-0.02	201	B:ASP 201		85.00	0.12	|	-0.00
202	A:ILE 202		40.92	0.00		0.00	202	B:ILE 202		40.29	0.00		0.00
203	A:LEU 203		83.97	9.24	||	0.15	203	B:LEU 203		83.57	9.53	||	0.15
204	A:SER 204		15.17	7.47	|||||	-0.09	204	B:SER 204		14.22	6.98	|||||	-0.08
205	A:TRP 205		7.22	0.48	|	0.01	205	B:TRP 205		7.54	0.48	|	0.01
206	A:SER 206		11.55	0.00		0.00	206	B:SER 206		11.55	0.00		0.00
207	A:ARG 207		89.67	51.48	||||||	-0.41	207	B:ARG 207		89.77	52.76	||||||	-0.46
208	A:HIS 208		107.26	76.01	||||||||	-0.45	208	B:HIS 208		106.84	73.98	|||||||	-0.42
209	A:HIS 209		75.65	53.84	||||||||	0.04	209	B:HIS 209		76.54	52.37	|||||||	0.08
210	A:ARG 210		17.49	0.00		0.00	210	B:ARG 210		16.03	0.00		0.00
211	A:VAL 211		21.49	0.00		0.00	211	B:VAL 211		21.49	0.00		0.00
212	A:PHE 212		7.20	0.00		0.00	212	B:PHE 212		7.20	0.00		0.00
213	A:PRO 213		3.80	0.00		0.00	213	B:PRO 213		3.74	0.00		0.00
214	A:GLY 214		26.10	0.00		0.00	214	B:GLY 214		26.00	0.00		0.00
215	A:GLU 215		19.27	0.00		0.00	215	B:GLU 215		19.10	0.00		0.00
216	A:GLY 216		13.56	0.00		0.00	216	B:GLY 216		13.61	0.00		0.00
217	A:ASP 217		34.31	0.00		0.00	217	B:ASP 217		33.29	0.00		0.00
218	A:PHE 218		37.39	0.00		0.00	218	B:PHE 218		36.78	0.00		0.00
219	A:ASP 219		76.56	0.00		0.00	219	B:ASP 219		77.72	0.00		0.00
220	A:LEU 220		8.02	0.00		0.00	220	B:LEU 220		7.01	0.00		0.00
221	A:VAL 221		27.44	0.00		0.00	221	B:VAL 221		25.75	0.00		0.00
222	A:LYS 222		59.99	0.00		0.00	222	B:LYS 222		60.97	0.00		0.00
223	A:PHE 223		0.15	0.00		0.00	223	B:PHE 223		0.00	0.00		0.00
224	A:MET 224		9.58	0.00		0.00	224	B:MET 224		9.69	0.00		0.00
225	A:VAL 225		10.56	0.00		0.00	225	B:VAL 225		10.38	0.00		0.00
226	A:HIS 226		7.27	0.00		0.00	226	B:HIS 226		6.84	0.00		0.00
227	A:LEU 227		2.84	0.00		0.00	227	B:LEU 227		2.84	0.00		0.00
228	A:ALA 228		38.34	0.00		0.00	228	B:ALA 228		37.55	0.00		0.00
229	A:LYS 229		51.10	0.00		0.00	229	B:LYS 229		53.15	0.00		0.00
230	A:THR 230		21.97	0.00		0.00	230	B:THR 230		22.23	0.00		0.00
231	A:GLY 231		51.50	0.00		0.00	231	B:GLY 231		51.78	0.00		0.00
232	A:TYR 232		20.58	0.00		0.00	232	B:TYR 232		21.44	0.00		0.00
233	A:ASP 233		106.75	0.00		0.00	233	B:ASP 233		104.69	0.00		0.00
234	A:GLY 234		14.68	0.00		0.00	234	B:GLY 234		15.05	0.00		0.00
235	A:PRO 235		27.60	0.00		0.00	235	B:PRO 235		27.62	0.00		0.00
236	A:ILE 236		10.44	0.00		0.00	236	B:ILE 236		10.05	0.00		0.00
237	A:SER 237		0.00	0.00		0.00	237	B:SER 237		0.00	0.00		0.00
238	A:LEU 238		0.00	0.00		0.00	238	B:LEU 238		0.00	0.00		0.00
239	A:GLU 239		16.39	0.00		0.00	239	B:GLU 239		15.57	0.00		0.00
240	A:ILE 240		15.90	0.00		0.00	240	B:ILE 240		15.40	0.00		0.00
241	A:PHE 241		85.61	7.35	|	0.12	241	B:PHE 241		83.59	6.88	|	0.11
242	A:ASN 242		32.09	0.00		0.00	242	B:ASN 242		31.23	0.00		0.00
243	A:ASP 243		70.97	6.05	|	-0.04	243	B:ASP 243		70.34	4.29	|	-0.04
244	A:SER 244		89.82	52.05	||||||	0.30	244	B:SER 244		90.33	52.98	||||||	0.32
245	A:PHE 245		194.66	169.87	|||||||||	2.58	245	B:PHE 245		195.14	167.80	|||||||||	2.57
246	A:ARG 246	H	134.81	101.50	||||||||	-0.47	246	B:ARG 246	H	133.08	100.53	||||||||	-0.54
247	A:LYS 247	HS	111.92	94.43	|||||||||	1.37	247	B:LYS 247	HS	113.42	97.43	|||||||||	1.41
248	A:ALA 248		25.61	2.88	||	-0.03	248	B:ALA 248		24.65	2.41	|	-0.03
249	A:GLU 249	HS	62.76	60.23	||||||||||	0.78	249	B:GLU 249	HS	62.65	59.83	||||||||||	0.76
250	A:VAL 250		26.46	12.94	|||||	0.20	250	B:VAL 250		26.13	11.43	|||||	0.18
251	A:GLY 251		27.59	11.60	|||||	0.14	251	B:GLY 251		27.65	11.31	|||||	0.12
252	A:ARG 252	H	90.32	75.82	|||||||||	0.56	252	B:ARG 252	H	90.34	75.19	|||||||||	0.54
253	A:THR 253		19.34	0.00		0.00	253	B:THR 253		18.62	0.00		0.00
254	A:ALA 254		0.00	0.00		0.00	254	B:ALA 254		0.00	0.00		0.00
255	A:ILE 255		48.50	10.61	|||	0.17	255	B:ILE 255		48.38	9.27	||	0.15

256	A:ASP 256	11.34	0.00	0.00	256	B:ASP 256	12.33	0.00	0.00
257	A:GLY 257	0.00	0.00	0.00	257	B:GLY 257	0.00	0.00	0.00
258	A:LEU 258	30.10	0.00	0.00	258	B:LEU 258	29.94	0.00	0.00
259	A:ARG 259	37.28	0.00	0.00	259	B:ARG 259	37.21	0.00	0.00
260	A:SER 260	0.00	0.00	0.00	260	B:SER 260	0.00	0.00	0.00
261	A:LEU 261	0.00	0.00	0.00	261	B:LEU 261	0.00	0.00	0.00
262	A:ARG 262	34.03	0.00	0.00	262	B:ARG 262	34.77	0.00	0.00
263	A:TRP 263	22.92	0.00	0.00	263	B:TRP 263	23.07	0.00	0.00
264	A:LEU 264	6.34	0.00	0.00	264	B:LEU 264	6.65	0.00	0.00
265	A:GLU 265	3.65	0.00	0.00	265	B:GLU 265	3.16	0.00	0.00
266	A:ASP 266	3.44	0.00	0.00	266	B:ASP 266	3.44	0.00	0.00
267	A:GLN 267	62.13	0.00	0.00	267	B:GLN 267	60.44	0.00	0.00
268	A:THR 268	2.34	0.00	0.00	268	B:THR 268	2.34	0.00	0.00
269	A:TRP 269	71.54	0.00	0.00	269	B:TRP 269	70.59	0.00	0.00
270	A:HIS 270	79.48	0.00	0.00	270	B:HIS 270	77.98	0.00	0.00
271	A:ALA 271	31.42	0.00	0.00	271	B:ALA 271	31.25	0.00	0.00
272	A:LEU 272	2.68	0.00	0.00	272	B:LEU 272	2.51	0.00	0.00
273	A:ASN 273	62.76	0.00	0.00	273	B:ASN 273	64.05	0.00	0.00
274	A:ALA 274	91.95	0.00	0.00	274	B:ALA 274	90.90	0.00	0.00
275	A:GLU 275	68.84	0.00	0.00	275	B:GLU 275	67.37	0.00	0.00
276	A:ASP 276	97.84	0.00	0.00	276	B:ASP 276	97.98	0.00	0.00
277	A:ARG 277	100.21	0.00	0.00	277	B:ARG 277	101.40	0.00	0.00
278	A:PRO 278	81.02	0.00	0.00	278	B:PRO 278	81.01	0.00	0.00
279	A:SER 279	58.30	0.00	0.00	279	B:SER 279	57.04	0.00	0.00
280	A:ALA 280	88.76	0.00	0.00	280	B:ALA 280	89.75	0.00	0.00
281	A:LEU 281	149.78	0.00	0.00	281	B:LEU 281	147.84	0.00	0.00
282	A:GLU 282	73.03	0.00	0.00	282	B:GLU 282	72.08	0.00	0.00
283	A:LEU 283	17.47	0.00	0.00	283	B:LEU 283	19.02	0.00	0.00
284	A:ARG 284	67.93	0.00	0.00	284	B:ARG 284	68.79	0.00	0.00
285	A:ALA 285	75.47	0.00	0.00	285	B:ALA 285	77.14	0.00	0.00
286	A:LEU 286	22.74	0.00	0.00	286	B:LEU 286	23.45	0.00	0.00
287	A:PRO 287	50.56	0.00	0.00	287	B:PRO 287	50.63	0.00	0.00
288	A:GLU 288	102.40	0.00	0.00	288	B:GLU 288	102.45	0.00	0.00
289	A:VAL 289	25.11	0.00	0.00	289	B:VAL 289	25.14	0.00	0.00
290	A:ALA 290	6.85	0.00	0.00	290	B:ALA 290	7.35	0.00	0.00
291	A:GLU 291	71.86	0.00	0.00	291	B:GLU 291	72.30	0.00	0.00
292	A:PRO 292	1.96	0.00	0.00	292	B:PRO 292	2.53	0.00	0.00
293	A:GLU 293	109.10	0.00	0.00	293	B:GLU 293	111.14	0.00	0.00
294	A:GLY 294	9.54	0.00	0.00	294	B:GLY 294	9.71	0.00	0.00
295	A:VAL 295	17.68	0.00	0.00	295	B:VAL 295	18.20	0.00	0.00
296	A:ASP 296	32.69	0.00	0.00	296	B:ASP 296	33.11	0.00	0.00
297	A:PHE 297	18.93	0.00	0.00	297	B:PHE 297	18.16	0.00	0.00
298	A:ILE 298	15.57	0.00	0.00	298	B:ILE 298	14.90	0.00	0.00
299	A:GLU 299	23.33	0.00	0.00	299	B:GLU 299	23.24	0.00	0.00
300	A:ILE 300	17.08	0.00	0.00	300	B:ILE 300	18.73	0.00	0.00
301	A:ALA 301	1.42	0.00	0.00	301	B:ALA 301	1.66	0.00	0.00
302	A:THR 302	26.73	0.00	0.00	302	B:THR 302	27.05	0.00	0.00
303	A:GLY 303	42.11	0.00	0.00	303	B:GLY 303	41.81	0.00	0.00
304	A:ARG 304	150.11	0.00	0.00	304	B:ARG 304	152.51	0.00	0.00
305	A:LEU 305	90.70	0.00	0.00	305	B:LEU 305	90.42	0.00	0.00
306	A:GLY 306	33.69	0.00	0.00	306	B:GLY 306	32.81	0.00	0.00
307	A:GLU 307	94.21	0.00	0.00	307	B:GLU 307	92.19	0.00	0.00
308	A:THR 308	11.57	0.00	0.00	308	B:THR 308	10.86	0.00	0.00
309	A:ILE 309	29.87	0.00	0.00	309	B:ILE 309	29.96	0.00	0.00
310	A:ARG 310	113.19	0.00	0.00	310	B:ARG 310	114.19	0.00	0.00
311	A:VAL 311	11.22	0.00	0.00	311	B:VAL 311	12.06	0.00	0.00
312	A:LEU 312	7.86	0.00	0.00	312	B:LEU 312	8.19	0.00	0.00
313	A:HIS 313	58.72	0.00	0.00	313	B:HIS 313	58.16	0.00	0.00
314	A:GLN 314	25.80	0.00	0.00	314	B:GLN 314	25.74	0.00	0.00
315	A:LEU 315	3.56	0.00	0.00	315	B:LEU 315	3.11	0.00	0.00
316	A:GLY 316	2.17	0.00	0.00	316	B:GLY 316	2.65	0.00	0.00
317	A:PHE 317	1.23	0.00	0.00	317	B:PHE 317	1.35	0.00	0.00
318	A:ARG 318	50.64	0.00	0.00	318	B:ARG 318	51.35	0.00	0.00
319	A:LEU 319	54.80	0.00	0.00	319	B:LEU 319	53.96	0.00	0.00
320	A:GLY 320	4.17	0.00	0.00	320	B:GLY 320	3.84	0.00	0.00
321	A:GLY 321	0.00	0.00	0.00	321	B:GLY 321	0.34	0.00	0.00
322	A:HIS 322	74.37	0.00	0.00	322	B:HIS 322	72.50	0.00	0.00
323	A:HIS 323	30.13	0.00	0.00	323	B:HIS 323	30.06	0.00	0.00
324	A:CYS 324	77.18	0.00	0.00	324	B:CYS 324	76.82	0.00	0.00
325	A:SER 325	99.12	0.00	0.00	325	B:SER 325	98.94	0.00	0.00
326	A:LYS 326	47.16	0.00	0.00	326	B:LYS 326	46.95	0.00	0.00
327	A:GLN 327	63.00	0.00	0.00	327	B:GLN 327	62.66	0.00	0.00
328	A:ASP 328	36.12	0.00	0.00	328	B:ASP 328	36.17	0.00	0.00
329	A:TYR 329	11.90	0.00	0.00	329	B:TYR 329	12.48	0.00	0.00
330	A:GLN 330	33.74	0.00	0.00	330	B:GLN 330	34.17	0.00	0.00
331	A:VAL 331	14.66	0.00	0.00	331	B:VAL 331	15.06	0.00	0.00
332	A:TRP 332	9.97	0.00	0.00	332	B:TRP 332	10.51	0.00	0.00
333	A:THR 333	18.42	0.00	0.00	333	B:THR 333	18.38	0.00	0.00
334	A:GLN 334	0.12	0.00	0.00	334	B:GLN 334	0.25	0.00	0.00
335	A:GLY 335	24.19	0.00	0.00	335	B:GLY 335	24.68	0.00	0.00
336	A:ASP 336	103.83	0.00	0.00	336	B:ASP 336	105.50	0.00	0.00

337	A:VAL 337		2.18	0.00	0.00	337	B:VAL 337		2.68	0.00	0.00
338	A:ARG 338		35.51	0.00	0.00	338	B:ARG 338		35.84	0.00	0.00
339	A:ILE 339		12.72	0.00	0.00	339	B:ILE 339		13.89	0.00	0.00
340	A:VAL 340		10.04	0.00	0.00	340	B:VAL 340		10.21	0.00	0.00
341	A:VAL 341		5.13	0.00	0.00	341	B:VAL 341		5.76	0.00	0.00
342	A:CYS 342		7.28	0.00	0.00	342	B:CYS 342		7.24	0.00	0.00
343	A:ASP 343		14.61	0.00	0.00	343	B:ASP 343		14.74	0.00	0.00
344	A:ARG 344		62.09	0.00	0.00	344	B:ARG 344		63.68	0.00	0.00
345	A:GLY 345		59.53	2.33 |	-0.03	345	B:GLY 345		59.91	1.36 |	-0.02
346	A:VAL 346		156.62	54.43 ||||	0.87	346	B:VAL 346		156.38	56.53 ||||	0.90
347	A:THR 347		74.96	0.00	0.00	347	B:THR 347		74.98	0.00	0.00
348	A:GLY 348		56.75	16.07 |||	0.26	348	B:GLY 348		56.53	17.58 ||||	0.28
349	A:ALA 349		60.97	6.19 ||	0.01	349	B:ALA 349		61.87	6.38 ||	0.01
350	A:PRO 350		96.97	50.11 |||||	0.80	350	B:PRO 350		96.48	48.48 |||||	0.78
351	A:THR 351		59.28	0.00	0.00	351	B:THR 351		58.88	0.00	0.00
352	A:THR 352		6.94	0.00	0.00	352	B:THR 352		7.20	0.00	0.00
353	A:ILE 353		14.62	0.00	0.00	353	B:ILE 353		14.38	0.00	0.00
354	A:SER 354		30.48	0.00	0.00	354	B:SER 354		30.23	0.00	0.00
355	A:ALA 355		12.70	0.00	0.00	355	B:ALA 355		12.94	0.00	0.00
356	A:MET 356		24.80	0.00	0.00	356	B:MET 356		23.65	0.00	0.00
357	A:GLY 357		0.00	0.00	0.00	357	B:GLY 357		0.00	0.00	0.00
358	A:PHE 358		5.47	0.00	0.00	358	B:PHE 358		6.10	0.00	0.00
359	A:ASP 359		39.08	0.00	0.00	359	B:ASP 359		38.92	0.00	0.00
360	A:THR 360		0.73	0.00	0.00	360	B:THR 360		0.15	0.00	0.00
361	A:PRO 361		61.10	0.00	0.00	361	B:PRO 361		62.23	0.00	0.00
362	A:ASP 362		64.51	0.00	0.00	362	B:ASP 362		61.91	0.00	0.00
363	A:PRO 363		30.74	0.00	0.00	363	B:PRO 363		32.38	0.00	0.00
364	A:GLU 364		90.10	0.00	0.00	364	B:GLU 364		90.35	0.00	0.00
365	A:ALA 365		23.78	0.00	0.00	365	B:ALA 365		23.15	0.00	0.00
366	A:ALA 366		0.33	0.00	0.00	366	B:ALA 366		0.33	0.00	0.00
367	A:HIS 367		32.22	0.00	0.00	367	B:HIS 367		32.98	0.00	0.00
368	A:ALA 368		36.18	0.00	0.00	368	B:ALA 368		35.83	0.00	0.00
369	A:ARG 369		5.99	0.00	0.00	369	B:ARG 369		5.72	0.00	0.00
370	A:ALA 370		0.50	0.00	0.00	370	B:ALA 370		0.50	0.00	0.00
371	A:GLU 371		72.03	0.00	0.00	371	B:GLU 371		72.97	0.00	0.00
372	A:LEU 372		107.10	0.00	0.00	372	B:LEU 372		107.05	0.00	0.00
373	A:LEU 373		5.67	0.00	0.00	373	B:LEU 373		4.34	0.00	0.00
374	A:ARG 374		114.16	0.00	0.00	374	B:ARG 374		114.68	0.00	0.00
375	A:ALA 375		19.09	0.00	0.00	375	B:ALA 375		19.39	0.00	0.00
376	A:GLN 376	H	91.98	44.23 |||||	-0.48	376	B:GLN 376	H	91.18	46.41 |||||	-0.48
377	A:THR 377		28.31	0.00	0.00	377	B:THR 377		27.50	0.00	0.00
378	A:ILE 378		4.93	0.00	0.00	378	B:ILE 378		4.26	0.00	0.00
379	A:ASP 379		70.66	0.00	0.00	379	B:ASP 379		71.31	0.00	0.00
380	A:ARG 380		107.22	0.00	0.00	380	B:ARG 380		105.60	0.00	0.00
381	A:PRO 381		107.85	0.00	0.00	381	B:PRO 381		109.15	0.00	0.00
382	A:HIS 382		83.95	24.55 |||	0.10	382	B:HIS 382		84.20	24.19 |||	0.08
383	A:ILE 383		148.68	88.61 |||||	0.81	383	B:ILE 383		148.76	87.11 |||||	0.80
384	A:GLU 384		79.06	29.44 ||||	0.38	384	B:GLU 384		78.00	30.52 ||||	0.40
385	A:GLY 385		4.07	0.00	0.00	385	B:GLY 385		4.52	0.00	0.00
386	A:GLU 386		99.14	0.00	0.00	386	B:GLU 386		97.51	0.00	0.00
387	A:VAL 387		24.71	0.00	0.00	387	B:VAL 387		24.07	0.00	0.00
388	A:ASP 388		64.56	0.00	0.00	388	B:ASP 388		65.89	0.00	0.00
389	A:LEU 389		53.73	0.00	0.00	389	B:LEU 389		53.77	0.00	0.00
390	A:LYS 390		34.99	0.00	0.00	390	B:LYS 390		35.32	0.00	0.00
391	A:GLY 391		2.52	0.00	0.00	391	B:GLY 391		2.40	0.00	0.00
392	A:VAL 392		2.33	0.00	0.00	392	B:VAL 392		2.00	0.00	0.00
393	A:TYR 393		84.43	16.88 ||	0.23	393	B:TYR 393		84.55	16.99 |||	0.23
394	A:ALA 394		7.86	0.00	0.00	394	B:ALA 394		6.86	0.00	0.00
395	A:PRO 395		38.80	4.86 ||	0.08	395	B:PRO 395		39.54	5.03 ||	0.08
396	A:ASP 396	H	82.93	66.97 ||||||||	0.22	396	B:ASP 396	H	81.96	65.63 ||||||||	0.20
397	A:GLY 397		43.17	6.77 ||	0.07	397	B:GLY 397		42.90	7.63 ||	0.06
398	A:VAL 398		40.95	0.00	0.00	398	B:VAL 398		40.87	0.00	0.00
399	A:GLU 399		18.96	0.00	0.00	399	B:GLU 399		17.93	0.00	0.00
400	A:LEU 400		17.91	0.00	0.00	400	B:LEU 400		17.41	0.00	0.00
401	A:PHE 401		5.06	0.00	0.00	401	B:PHE 401		5.22	0.00	0.00
402	A:PHE 402		3.55	0.00	0.00	402	B:PHE 402		3.57	0.00	0.00
403	A:ALA 403		4.35	0.00	0.00	403	B:ALA 403		4.35	0.00	0.00
404	A:GLY 404		12.02	0.00	0.00	404	B:GLY 404		11.80	0.00	0.00
405	A:PRO 405		96.74	0.00	0.00	405	B:PRO 405		98.98	0.00	0.00
406	A:SER 406		66.50	0.00	0.00	406	B:SER 406		65.17	0.00	0.00
407	A:PRO 407		87.05	0.00	0.00	407	B:PRO 407		86.34	0.00	0.00
408	A:ASP 408		146.94	0.00	0.00	408	B:ASP 408		147.21	0.00	0.00
409	A:GLY 409		47.87	0.00	0.00	409	B:GLY 409		47.57	0.00	0.00
410	A:MET 410		127.26	0.00	0.00	410	B:MET 410		126.51	0.00	0.00
411	A:PRO 411		9.59	0.00	0.00	411	B:PRO 411		10.60	0.00	0.00
412	A:GLU 412		51.55	0.00	0.00	412	B:GLU 412		51.55	0.00	0.00
413	A:TRP 413		83.88	0.00	0.00	413	B:TRP 413		85.08	0.00	0.00
414	A:LEU 414		58.66	0.00	0.00	414	B:LEU 414		58.23	0.00	0.00
415	A:PRO 415		75.97	0.00	0.00	415	B:PRO 415		74.80	0.00	0.00
416	A:GLU 416		38.85	0.00	0.00	416	B:GLU 416		37.73	0.00	0.00
417	A:PHE 417		85.48	0.00	0.00	417	B:PHE 417		86.22	0.00	0.00

418	A:GLY 418		37.46	0.00	0.00	418	B:GLY 418		37.03	0.00	0.00
419	A:VAL 419		72.35	0.00	0.00	419	B:VAL 419		70.51	0.00	0.00
420	A:GLU 420		69.66	0.00	0.00	420	B:GLU 420		68.47	0.00	0.00
421	A:LYS 421		162.24	0.00	0.00	421	B:LYS 421		162.53	0.00	0.00
422	A:GLN 422		88.81	0.00	0.00	422	B:GLN 422		88.12	0.00	0.00
423	A:GLU 423		76.63	0.00	0.00	423	B:GLU 423		75.78	0.00	0.00
424	A:ALA 424		48.65	0.00	0.00	424	B:ALA 424		49.22	0.00	0.00
425	A:GLY 425		27.39	0.00	0.00	425	B:GLY 425		26.03	0.00	0.00
426	A:LEU 426		94.66	0.00	0.00	426	B:LEU 426		93.92	0.00	0.00
427	A:ILE 427		1.44	0.00	0.00	427	B:ILE 427		1.52	0.00	0.00
428	A:GLU 428		54.11	0.00	0.00	428	B:GLU 428		53.27	0.00	0.00
429	A:ALA 429		50.99	0.00	0.00	429	B:ALA 429		50.79	0.00	0.00
430	A:ILE 430		17.43	0.00	0.00	430	B:ILE 430		17.91	0.00	0.00
431	A:ASP 431		19.85	0.00	0.00	431	B:ASP 431		19.66	0.00	0.00
432	A:HIS 432		1.00	0.00	0.00	432	B:HIS 432		1.17	0.00	0.00
433	A:VAL 433		14.79	0.00	0.00	433	B:VAL 433		14.83	0.00	0.00
434	A:ASN 434		2.95	0.00	0.00	434	B:ASN 434		2.38	0.00	0.00
435	A:PHE 435		32.81	2.20	0.04	435	B:PHE 435		31.40	2.04	0.03
436	A:ALA 436		14.71	0.00	0.00	436	B:ALA 436		14.37	0.00	0.00
437	A:GLN 437		15.77	5.59	0.02	437	B:GLN 437		14.90	5.01	0.02
438	A:PRO 438		24.68	10.20	0.16	438	B:PRO 438		24.18	11.29	0.18
439	A:TRP 439		162.45	104.73	1.60	439	B:TRP 439		165.14	109.72	1.73
440	A:GLN 440	H	49.15	12.78	0.19	440	B:GLN 440	H	50.57	13.28	0.19
441	A:HIS 441		37.94	35.76	1.20	441	B:HIS 441		37.56	35.30	1.21
442	A:PHE 442		28.33	0.00	0.00	442	B:PHE 442		28.16	0.00	0.00
443	A:ASP 443		39.33	0.00	0.00	443	B:ASP 443		38.23	0.00	0.00
444	A:GLU 444		28.47	0.24	-0.00	444	B:GLU 444		27.36	0.37	-0.00
445	A:ALA 445		2.02	0.00	0.00	445	B:ALA 445		2.66	0.00	0.00
446	A:VAL 446		10.21	0.00	0.00	446	B:VAL 446		10.22	0.00	0.00
447	A:LEU 447		0.00	0.00	0.00	447	B:LEU 447		0.00	0.00	0.00
448	A:PHE 448		12.34	0.00	0.00	448	B:PHE 448		11.88	0.00	0.00
449	A:TYR 449		30.19	0.00	0.00	449	B:TYR 449		30.20	0.00	0.00
450	A:THR 450		8.78	0.00	0.00	450	B:THR 450		8.62	0.00	0.00
451	A:ALA 451		0.00	0.00	0.00	451	B:ALA 451		0.00	0.00	0.00
452	A:LEU 452		4.48	0.00	0.00	452	B:LEU 452		5.58	0.00	0.00
453	A:MET 453		5.70	0.00	0.00	453	B:MET 453		5.54	0.00	0.00
454	A:ALA 454		24.35	0.00	0.00	454	B:ALA 454		24.55	0.00	0.00
455	A:LEU 455		1.97	0.00	0.00	455	B:LEU 455		2.63	0.00	0.00
456	A:GLU 456		74.99	0.00	0.00	456	B:GLU 456		74.80	0.00	0.00
457	A:THR 457		77.68	0.00	0.00	457	B:THR 457		76.97	0.00	0.00
458	A:VAL 458		39.23	0.00	0.00	458	B:VAL 458		39.26	0.00	0.00
459	A:ARG 459		80.44	0.00	0.00	459	B:ARG 459		78.26	0.00	0.00
460	A:GLU 460		34.98	0.00	0.00	460	B:GLU 460		33.99	0.00	0.00
461	A:ASP 461		85.13	0.00	0.00	461	B:ASP 461		85.50	0.00	0.00
462	A:GLU 462		42.43	0.00	0.00	462	B:GLU 462		44.77	0.00	0.00
463	A:PHE 463		170.23	0.00	0.00	463	B:PHE 463		172.74	0.00	0.00
464	A:PRO 464		19.83	0.00	0.00	464	B:PRO 464		19.38	0.00	0.00
465	A:SER 465		70.32	26.85	0.02	465	B:SER 465		70.53	27.29	0.05
466	A:PRO 466	H	144.03	27.56	-0.19	466	B:PRO 466	H	145.01	28.57	-0.18
467	A:ILE 467		122.47	7.67	0.11	467	B:ILE 467		122.94	7.96	0.13
468	A:GLY 468		36.83	23.26	0.16	468	B:GLY 468		36.84	22.78	0.15
469	A:LEU 469		71.22	0.00	0.00	469	B:LEU 469		72.12	0.00	0.00
470	A:VAL 470		87.59	1.67	0.03	470	B:VAL 470		88.01	1.00	0.02
471	A:ARG 471		68.56	0.00	0.00	471	B:ARG 471		67.75	0.00	0.00
472	A:ASN 472		33.49	0.00	0.00	472	B:ASN 472		33.85	0.00	0.00
473	A:GLN 473		46.72	0.00	0.00	473	B:GLN 473		47.94	0.00	0.00
474	A:VAL 474		11.24	0.00	0.00	474	B:VAL 474		11.67	0.00	0.00
475	A:MET 475		3.34	0.00	0.00	475	B:MET 475		3.18	0.00	0.00
476	A:ARG 476		56.55	0.00	0.00	476	B:ARG 476		56.13	0.00	0.00
477	A:SER 477		0.29	0.00	0.00	477	B:SER 477		0.17	0.00	0.00
478	A:PRO 478		56.66	0.00	0.00	478	B:PRO 478		57.77	0.00	0.00
479	A:ASN 479		61.59	0.00	0.00	479	B:ASN 479		61.35	0.00	0.00
480	A:ASP 480		49.10	0.00	0.00	480	B:ASP 480		47.89	0.00	0.00
481	A:ALA 481		31.93	0.00	0.00	481	B:ALA 481		32.67	0.00	0.00
482	A:VAL 482		13.40	0.00	0.00	482	B:VAL 482		13.38	0.00	0.00
483	A:ARG 483		13.08	0.00	0.00	483	B:ARG 483		12.24	0.00	0.00
484	A:LEU 484		6.37	0.00	0.00	484	B:LEU 484		6.03	0.00	0.00
485	A:LEU 485		12.56	0.00	0.00	485	B:LEU 485		12.71	0.00	0.00
486	A:LEU 486		3.69	0.00	0.00	486	B:LEU 486		3.99	0.00	0.00
487	A:SER 487		19.45	0.00	0.00	487	B:SER 487		20.22	0.00	0.00
488	A:VAL 488		68.10	5.52	0.09	488	B:VAL 488		68.57	6.37	0.10
489	A:ALA 489		4.29	0.00	0.00	489	B:ALA 489		4.41	0.00	0.00
490	A:PRO 490		72.26	0.00	0.00	490	B:PRO 490		70.88	0.00	0.00
491	A:GLU 491		73.54	30.85	0.15	491	B:GLU 491		72.55	30.73	0.12
492	A:ASP 492		73.21	41.08	0.00	492	B:ASP 492		72.97	41.15	0.01
493	A:GLY 493		77.34	14.48	0.19	493	B:GLY 493		78.89	16.01	0.20
494	A:GLU 494		162.48	92.01	-0.55	494	B:GLU 494		163.92	104.15	-0.54
495	A:GLN 495		60.16	11.30	-0.13	495	B:GLN 495		61.63	11.79	-0.13
496	A:GLY 496		42.11	12.73	0.20	496	B:GLY 496		42.37	13.57	0.22
497	A:ASP 497		152.70	94.53	-0.34	497	B:ASP 497		152.07	96.45	-0.38
498	A:PHE 498		93.54	0.33	0.01	498	B:PHE 498		94.87	1.34	0.02

<table>
<tr><td>499</td><td>A:LEU 499</td><td>78.53</td><td>71.83 |||||||||</td><td>0.91</td><td>499</td><td>B:LEU 499</td><td>79.21</td><td>73.86 |||||||||</td><td>0.92</td></tr>
<tr><td>500</td><td>A:ASN 500</td><td>44.77</td><td>14.98 ||||</td><td>0.12</td><td>500</td><td>B:ASN 500</td><td>44.96</td><td>14.99 ||||</td><td>0.12</td></tr>
<tr><td>501</td><td>A:ALA 501</td><td>51.66</td><td>37.83 |||||||</td><td>0.59</td><td>501</td><td>B:ALA 501</td><td>51.75</td><td>38.54 |||||||</td><td>0.61</td></tr>
<tr><td>502</td><td>A:ALA 502</td><td>31.23</td><td>24.25 |||||||</td><td>-0.02</td><td>502</td><td>B:ALA 502</td><td>31.35</td><td>24.42 |||||||</td><td>-0.01</td></tr>
<tr><td>503</td><td>A:TYR 503</td><td>42.22</td><td>15.29 ||||</td><td>-0.01</td><td>503</td><td>B:TYR 503</td><td>42.69</td><td>14.38 ||||</td><td>-0.02</td></tr>
<tr><td>504</td><td>A:PRO 504</td><td>5.54</td><td>0.00</td><td>0.00</td><td>504</td><td>B:PRO 504</td><td>4.79</td><td>0.00</td><td>0.00</td></tr>
<tr><td>505</td><td>A:GLU 505</td><td>33.06</td><td>0.00</td><td>0.00</td><td>505</td><td>B:GLU 505</td><td>31.40</td><td>0.00</td><td>0.00</td></tr>
<tr><td>506</td><td>A:HIS 506</td><td>0.00</td><td>0.00</td><td>0.00</td><td>506</td><td>B:HIS 506</td><td>0.00</td><td>0.00</td><td>0.00</td></tr>
<tr><td>507</td><td>A:ILE 507</td><td>12.88</td><td>0.00</td><td>0.00</td><td>507</td><td>B:ILE 507</td><td>12.05</td><td>0.00</td><td>0.00</td></tr>
<tr><td>508</td><td>A:ALA 508</td><td>0.12</td><td>0.00</td><td>0.00</td><td>508</td><td>B:ALA 508</td><td>0.12</td><td>0.00</td><td>0.00</td></tr>
<tr><td>509</td><td>A:LEU 509</td><td>3.34</td><td>0.00</td><td>0.00</td><td>509</td><td>B:LEU 509</td><td>4.02</td><td>0.00</td><td>0.00</td></tr>
<tr><td>510</td><td>A:ALA 510</td><td>21.34</td><td>0.00</td><td>0.00</td><td>510</td><td>B:ALA 510</td><td>21.34</td><td>0.00</td><td>0.00</td></tr>
<tr><td>511</td><td>A:THR 511</td><td>12.06</td><td>0.00</td><td>0.00</td><td>511</td><td>B:THR 511</td><td>12.37</td><td>0.00</td><td>0.00</td></tr>
<tr><td>512</td><td>A:ALA 512</td><td>73.07</td><td>0.00</td><td>0.00</td><td>512</td><td>B:ALA 512</td><td>73.77</td><td>0.00</td><td>0.00</td></tr>
<tr><td>513</td><td>A:ASP 513</td><td>49.92</td><td>0.00</td><td>0.00</td><td>513</td><td>B:ASP 513</td><td>50.43</td><td>0.00</td><td>0.00</td></tr>
<tr><td>514</td><td>A:ILE 514</td><td>0.17</td><td>0.00</td><td>0.00</td><td>514</td><td>B:ILE 514</td><td>0.00</td><td>0.00</td><td>0.00</td></tr>
<tr><td>515</td><td>A:VAL 515</td><td>59.53</td><td>0.00</td><td>0.00</td><td>515</td><td>B:VAL 515</td><td>59.36</td><td>0.00</td><td>0.00</td></tr>
<tr><td>516</td><td>A:ALA 516</td><td>49.77</td><td>0.00</td><td>0.00</td><td>516</td><td>B:ALA 516</td><td>49.23</td><td>0.00</td><td>0.00</td></tr>
<tr><td>517</td><td>A:VAL 517</td><td>5.70</td><td>0.00</td><td>0.00</td><td>517</td><td>B:VAL 517</td><td>5.70</td><td>0.00</td><td>0.00</td></tr>
<tr><td>518</td><td>A:ALA 518</td><td>1.00</td><td>0.00</td><td>0.00</td><td>518</td><td>B:ALA 518</td><td>0.67</td><td>0.00</td><td>0.00</td></tr>
<tr><td>519</td><td>A:GLU 519</td><td>68.99</td><td>0.00</td><td>0.00</td><td>519</td><td>B:GLU 519</td><td>67.85</td><td>0.00</td><td>0.00</td></tr>
<tr><td>520</td><td>A:ARG 520</td><td>78.52</td><td>0.00</td><td>0.00</td><td>520</td><td>B:ARG 520</td><td>79.32</td><td>0.00</td><td>0.00</td></tr>
<tr><td>521</td><td>A:ALA 521</td><td>0.34</td><td>0.00</td><td>0.00</td><td>521</td><td>B:ALA 521</td><td>0.17</td><td>0.00</td><td>0.00</td></tr>
<tr><td>522</td><td>A:ARG 522</td><td>65.06</td><td>0.00</td><td>0.00</td><td>522</td><td>B:ARG 522</td><td>66.62</td><td>0.00</td><td>0.00</td></tr>
<tr><td>523</td><td>A:LYS 523</td><td>159.13</td><td>0.00</td><td>0.00</td><td>523</td><td>B:LYS 523</td><td>159.25</td><td>0.00</td><td>0.00</td></tr>
<tr><td>524</td><td>A:ARG 524</td><td>91.10</td><td>0.00</td><td>0.00</td><td>524</td><td>B:ARG 524</td><td>89.45</td><td>0.00</td><td>0.00</td></tr>
<tr><td>525</td><td>A:GLY 525</td><td>40.75</td><td>0.00</td><td>0.00</td><td>525</td><td>B:GLY 525</td><td>40.64</td><td>0.00</td><td>0.00</td></tr>
<tr><td>526</td><td>A:LEU 526</td><td>7.40</td><td>0.00</td><td>0.00</td><td>526</td><td>B:LEU 526</td><td>7.36</td><td>0.00</td><td>0.00</td></tr>
<tr><td>527</td><td>A:ASP 527</td><td>59.49</td><td>0.00</td><td>0.00</td><td>527</td><td>B:ASP 527</td><td>59.09</td><td>0.00</td><td>0.00</td></tr>
<tr><td>528</td><td>A:PHE 528</td><td>17.68</td><td>0.00</td><td>0.00</td><td>528</td><td>B:PHE 528</td><td>17.63</td><td>0.00</td><td>0.00</td></tr>
<tr><td>529</td><td>A:LEU 529</td><td>6.65</td><td>0.00</td><td>0.00</td><td>529</td><td>B:LEU 529</td><td>4.93</td><td>0.00</td><td>0.00</td></tr>
<tr><td>530</td><td>A:PRO 530</td><td>78.16</td><td>0.00</td><td>0.00</td><td>530</td><td>B:PRO 530</td><td>77.57</td><td>0.00</td><td>0.00</td></tr>
<tr><td>531</td><td>A:VAL 531</td><td>13.70</td><td>0.00</td><td>0.00</td><td>531</td><td>B:VAL 531</td><td>13.87</td><td>0.00</td><td>0.00</td></tr>
<tr><td>532</td><td>A:PRO 532</td><td>22.76</td><td>0.00</td><td>0.00</td><td>532</td><td>B:PRO 532</td><td>23.17</td><td>0.00</td><td>0.00</td></tr>
<tr><td>533</td><td>A:GLU 533</td><td>89.12</td><td>0.00</td><td>0.00</td><td>533</td><td>B:GLU 533</td><td>89.98</td><td>0.00</td><td>0.00</td></tr>
<tr><td>534</td><td>A:ASN 534</td><td>106.50</td><td>0.00</td><td>0.00</td><td>534</td><td>B:ASN 534</td><td>105.23</td><td>0.00</td><td>0.00</td></tr>
<tr><td>535</td><td>A:TYR 535</td><td>9.79</td><td>0.00</td><td>0.00</td><td>535</td><td>B:TYR 535</td><td>10.07</td><td>0.00</td><td>0.00</td></tr>
<tr><td>536</td><td>A:TYR 536</td><td>0.93</td><td>0.00</td><td>0.00</td><td>536</td><td>B:TYR 536</td><td>0.63</td><td>0.00</td><td>0.00</td></tr>
<tr><td>537</td><td>A:ASP 537</td><td>80.46</td><td>0.00</td><td>0.00</td><td>537</td><td>B:ASP 537</td><td>80.18</td><td>0.00</td><td>0.00</td></tr>
<tr><td>538</td><td>A:ASP 538</td><td>50.85</td><td>0.00</td><td>0.00</td><td>538</td><td>B:ASP 538</td><td>50.51</td><td>0.00</td><td>0.00</td></tr>
<tr><td>539</td><td>A:VAL 539</td><td>1.18</td><td>0.00</td><td>0.00</td><td>539</td><td>B:VAL 539</td><td>1.35</td><td>0.00</td><td>0.00</td></tr>
<tr><td>540</td><td>A:GLN 540</td><td>92.95</td><td>0.00</td><td>0.00</td><td>540</td><td>B:GLN 540</td><td>92.54</td><td>0.00</td><td>0.00</td></tr>
<tr><td>541</td><td>A:ALA 541</td><td>93.71</td><td>0.00</td><td>0.00</td><td>541</td><td>B:ALA 541</td><td>94.17</td><td>0.00</td><td>0.00</td></tr>
<tr><td>542</td><td>A:ARG 542</td><td>108.04</td><td>0.00</td><td>0.00</td><td>542</td><td>B:ARG 542</td><td>107.87</td><td>0.00</td><td>0.00</td></tr>
<tr><td>543</td><td>A:PHE 543</td><td>45.59</td><td>0.00</td><td>0.00</td><td>543</td><td>B:PHE 543</td><td>46.04</td><td>0.00</td><td>0.00</td></tr>
<tr><td>544</td><td>A:ASP 544</td><td>99.47</td><td>0.00</td><td>0.00</td><td>544</td><td>B:ASP 544</td><td>99.06</td><td>0.00</td><td>0.00</td></tr>
<tr><td>545</td><td>A:LEU 545</td><td>30.83</td><td>0.00</td><td>0.00</td><td>545</td><td>B:LEU 545</td><td>31.06</td><td>0.00</td><td>0.00</td></tr>
<tr><td>546</td><td>A:PRO 546</td><td>84.21</td><td>0.00</td><td>0.00</td><td>546</td><td>B:PRO 546</td><td>84.47</td><td>0.00</td><td>0.00</td></tr>
<tr><td>547</td><td>A:GLN 547</td><td>157.18</td><td>0.00</td><td>0.00</td><td>547</td><td>B:GLN 547</td><td>157.59</td><td>0.00</td><td>0.00</td></tr>
<tr><td>548</td><td>A:GLU 548</td><td>117.83</td><td>0.00</td><td>0.00</td><td>548</td><td>B:GLU 548</td><td>118.36</td><td>0.00</td><td>0.00</td></tr>
<tr><td>549</td><td>A:PHE 549</td><td>45.18</td><td>0.00</td><td>0.00</td><td>549</td><td>B:PHE 549</td><td>45.95</td><td>0.00</td><td>0.00</td></tr>
<tr><td>550</td><td>A:LEU 550</td><td>10.83</td><td>0.00</td><td>0.00</td><td>550</td><td>B:LEU 550</td><td>10.81</td><td>0.00</td><td>0.00</td></tr>
<tr><td>551</td><td>A:ASP 551</td><td>78.64</td><td>0.00</td><td>0.00</td><td>551</td><td>B:ASP 551</td><td>77.88</td><td>0.00</td><td>0.00</td></tr>
<tr><td>552</td><td>A:THR 552</td><td>58.29</td><td>0.00</td><td>0.00</td><td>552</td><td>B:THR 552</td><td>57.50</td><td>0.00</td><td>0.00</td></tr>
<tr><td>553</td><td>A:LEU 553</td><td>1.00</td><td>0.00</td><td>0.00</td><td>553</td><td>B:LEU 553</td><td>0.50</td><td>0.00</td><td>0.00</td></tr>
<tr><td>554</td><td>A:LYS 554</td><td>55.04</td><td>0.00</td><td>0.00</td><td>554</td><td>B:LYS 554</td><td>54.31</td><td>0.00</td><td>0.00</td></tr>
<tr><td>555</td><td>A:GLU 555</td><td>100.24</td><td>0.00</td><td>0.00</td><td>555</td><td>B:GLU 555</td><td>99.31</td><td>0.00</td><td>0.00</td></tr>
<tr><td>556</td><td>A:ASN 556</td><td>18.23</td><td>0.00</td><td>0.00</td><td>556</td><td>B:ASN 556</td><td>18.25</td><td>0.00</td><td>0.00</td></tr>
<tr><td>557</td><td>A:HIS 557</td><td>49.38</td><td>0.00</td><td>0.00</td><td>557</td><td>B:HIS 557</td><td>51.29</td><td>0.00</td><td>0.00</td></tr>
<tr><td>558</td><td>A:LEU 558</td><td>0.17</td><td>0.00</td><td>0.00</td><td>558</td><td>B:LEU 558</td><td>0.33</td><td>0.00</td><td>0.00</td></tr>
<tr><td>559</td><td>A:LEU 559</td><td>0.34</td><td>0.00</td><td>0.00</td><td>559</td><td>B:LEU 559</td><td>0.67</td><td>0.00</td><td>0.00</td></tr>
<tr><td>560</td><td>A:TYR 560</td><td>1.89</td><td>0.00</td><td>0.00</td><td>560</td><td>B:TYR 560</td><td>1.88</td><td>0.00</td><td>0.00</td></tr>
<tr><td>561</td><td>A:ASP 561</td><td>15.78</td><td>0.00</td><td>0.00</td><td>561</td><td>B:ASP 561</td><td>16.03</td><td>0.00</td><td>0.00</td></tr>
<tr><td>562</td><td>A:ARG 562</td><td>59.84</td><td>0.00</td><td>0.00</td><td>562</td><td>B:ARG 562</td><td>60.61</td><td>0.00</td><td>0.00</td></tr>
<tr><td>563</td><td>A:ASP 563</td><td>68.73</td><td>0.00</td><td>0.00</td><td>563</td><td>B:ASP 563</td><td>69.17</td><td>0.00</td><td>0.00</td></tr>
<tr><td>564</td><td>A:GLU 564</td><td>166.21</td><td>0.00</td><td>0.00</td><td>564</td><td>B:GLU 564</td><td>166.41</td><td>0.00</td><td>0.00</td></tr>
<tr><td>565</td><td>A:ASN 565</td><td>134.96</td><td>0.00</td><td>0.00</td><td>565</td><td>B:ASN 565</td><td>135.82</td><td>0.00</td><td>0.00</td></tr>
<tr><td>566</td><td>A:GLY 566</td><td>2.66</td><td>0.00</td><td>0.00</td><td>566</td><td>B:GLY 566</td><td>2.30</td><td>0.00</td><td>0.00</td></tr>
<tr><td>567</td><td>A:GLU 567</td><td>14.79</td><td>0.00</td><td>0.00</td><td>567</td><td>B:GLU 567</td><td>14.79</td><td>0.00</td><td>0.00</td></tr>
<tr><td>568</td><td>A:PHE 568</td><td>0.00</td><td>0.00</td><td>0.00</td><td>568</td><td>B:PHE 568</td><td>0.00</td><td>0.00</td><td>0.00</td></tr>
<tr><td>569</td><td>A:LEU 569</td><td>0.67</td><td>0.00</td><td>0.00</td><td>569</td><td>B:LEU 569</td><td>0.67</td><td>0.00</td><td>0.00</td></tr>
<tr><td>570</td><td>A:HIS 570</td><td>0.15</td><td>0.00</td><td>0.00</td><td>570</td><td>B:HIS 570</td><td>0.00</td><td>0.00</td><td>0.00</td></tr>
<tr><td>571</td><td>A:PHE 571</td><td>1.06</td><td>0.00</td><td>0.00</td><td>571</td><td>B:PHE 571</td><td>1.23</td><td>0.00</td><td>0.00</td></tr>
<tr><td>572</td><td>A:TYR 572</td><td>3.59</td><td>0.00</td><td>0.00</td><td>572</td><td>B:TYR 572</td><td>2.90</td><td>0.00</td><td>0.00</td></tr>
<tr><td>573</td><td>A:THR 573</td><td>7.67</td><td>0.00</td><td>0.00</td><td>573</td><td>B:THR 573</td><td>7.52</td><td>0.00</td><td>0.00</td></tr>
<tr><td>574</td><td>A:ARG 574</td><td>104.38</td><td>0.00</td><td>0.00</td><td>574</td><td>B:ARG 574</td><td>103.44</td><td>0.00</td><td>0.00</td></tr>
<tr><td>575</td><td>A:THR 575</td><td>52.42</td><td>0.00</td><td>0.00</td><td>575</td><td>B:THR 575</td><td>50.63</td><td>0.00</td><td>0.00</td></tr>
<tr><td>576</td><td>A:LEU 576</td><td>54.49</td><td>0.00</td><td>0.00</td><td>576</td><td>B:LEU 576</td><td>55.02</td><td>0.00</td><td>0.00</td></tr>
<tr><td>577</td><td>A:GLY 577</td><td>31.11</td><td>0.00</td><td>0.00</td><td>577</td><td>B:GLY 577</td><td>32.28</td><td>0.00</td><td>0.00</td></tr>
<tr><td>578</td><td>A:THR 578</td><td>36.46</td><td>0.00</td><td>0.00</td><td>578</td><td>B:THR 578</td><td>38.03</td><td>0.00</td><td>0.00</td></tr>
<tr><td>579</td><td>A:LEU 579</td><td>13.69</td><td>0.00</td><td>0.00</td><td>579</td><td>B:LEU 579</td><td>13.53</td><td>0.00</td><td>0.00</td></tr>
</table>

<table>
<tr><td>580</td><td>A:PHE 580</td><td></td><td>24.70</td><td>0.00</td><td>0.00</td><td>580</td><td>B:PHE 580</td><td></td><td>25.50</td><td>0.00</td><td>0.00</td></tr>
<tr><td>581</td><td>A:PHE 581</td><td></td><td>16.89</td><td>0.00</td><td>0.00</td><td>581</td><td>B:PHE 581</td><td></td><td>17.21</td><td>0.00</td><td>0.00</td></tr>
<tr><td>582</td><td>A:GLU 582</td><td></td><td>1.77</td><td>0.00</td><td>0.00</td><td>582</td><td>B:GLU 582</td><td></td><td>1.90</td><td>0.00</td><td>0.00</td></tr>
<tr><td>583</td><td>A:VAL 583</td><td></td><td>3.68</td><td>0.00</td><td>0.00</td><td>583</td><td>B:VAL 583</td><td></td><td>4.29</td><td>0.00</td><td>0.00</td></tr>
<tr><td>584</td><td>A:VAL 584</td><td></td><td>0.78</td><td>0.00</td><td>0.00</td><td>584</td><td>B:VAL 584</td><td></td><td>0.50</td><td>0.00</td><td>0.00</td></tr>
<tr><td>585</td><td>A:GLU 585</td><td></td><td>15.22</td><td>0.00</td><td>0.00</td><td>585</td><td>B:GLU 585</td><td></td><td>15.65</td><td>0.00</td><td>0.00</td></tr>
<tr><td>586</td><td>A:ARG 586</td><td></td><td>86.86</td><td>0.00</td><td>0.00</td><td>586</td><td>B:ARG 586</td><td></td><td>88.27</td><td>0.00</td><td>0.00</td></tr>
<tr><td>587</td><td>A:ARG 587</td><td></td><td>89.58</td><td>0.00</td><td>0.00</td><td>587</td><td>B:ARG 587</td><td></td><td>87.32</td><td>0.00</td><td>0.00</td></tr>
<tr><td>588</td><td>A:GLY 588</td><td></td><td>70.99</td><td>0.00</td><td>0.00</td><td>588</td><td>B:GLY 588</td><td></td><td>69.68</td><td>0.00</td><td>0.00</td></tr>
<tr><td>589</td><td>A:GLY 589</td><td></td><td>65.72</td><td>0.00</td><td>0.00</td><td>589</td><td>B:GLY 589</td><td></td><td>66.74</td><td>0.00</td><td>0.00</td></tr>
<tr><td>590</td><td>A:PHE 590</td><td></td><td>9.26</td><td>0.00</td><td>0.00</td><td>590</td><td>B:PHE 590</td><td></td><td>9.74</td><td>0.00</td><td>0.00</td></tr>
<tr><td>591</td><td>A:ALA 591</td><td></td><td>54.42</td><td>0.00</td><td>0.00</td><td>591</td><td>B:ALA 591</td><td></td><td>54.47</td><td>0.00</td><td>0.00</td></tr>
<tr><td>592</td><td>A:GLY 592</td><td></td><td>9.86</td><td>0.00</td><td>0.00</td><td>592</td><td>B:GLY 592</td><td></td><td>9.82</td><td>0.00</td><td>0.00</td></tr>
<tr><td>593</td><td>A:TRP 593</td><td></td><td>8.81</td><td>0.00</td><td>0.00</td><td>593</td><td>B:TRP 593</td><td></td><td>7.98</td><td>0.00</td><td>0.00</td></tr>
<tr><td>594</td><td>A:GLY 594</td><td></td><td>1.46</td><td>0.00</td><td>0.00</td><td>594</td><td>B:GLY 594</td><td></td><td>1.50</td><td>0.00</td><td>0.00</td></tr>
<tr><td>595</td><td>A:GLU 595</td><td></td><td>20.25</td><td>0.00</td><td>0.00</td><td>595</td><td>B:GLU 595</td><td></td><td>19.10</td><td>0.00</td><td>0.00</td></tr>
<tr><td>596</td><td>A:THR 596</td><td></td><td>14.48</td><td>0.00</td><td>0.00</td><td>596</td><td>B:THR 596</td><td></td><td>15.09</td><td>0.00</td><td>0.00</td></tr>
<tr><td>597</td><td>A:ASN 597</td><td></td><td>0.37</td><td>0.00</td><td>0.00</td><td>597</td><td>B:ASN 597</td><td></td><td>0.37</td><td>0.00</td><td>0.00</td></tr>
<tr><td>598</td><td>A:ALA 598</td><td></td><td>5.12</td><td>0.00</td><td>0.00</td><td>598</td><td>B:ALA 598</td><td></td><td>5.29</td><td>0.00</td><td>0.00</td></tr>
<tr><td>599</td><td>A:PRO 599</td><td></td><td>42.56</td><td>0.00</td><td>0.00</td><td>599</td><td>B:PRO 599</td><td></td><td>42.63</td><td>0.00</td><td>0.00</td></tr>
<tr><td>600</td><td>A:VAL 600</td><td></td><td>20.81</td><td>0.00</td><td>0.00</td><td>600</td><td>B:VAL 600</td><td></td><td>20.45</td><td>0.00</td><td>0.00</td></tr>
<tr><td>601</td><td>A:ARG 601</td><td></td><td>25.79</td><td>0.00</td><td>0.00</td><td>601</td><td>B:ARG 601</td><td></td><td>25.29</td><td>0.00</td><td>0.00</td></tr>
<tr><td>602</td><td>A:LEU 602</td><td></td><td>104.41</td><td>0.00</td><td>0.00</td><td>602</td><td>B:LEU 602</td><td></td><td>103.96</td><td>0.00</td><td>0.00</td></tr>
<tr><td>603</td><td>A:ALA 603</td><td></td><td>50.94</td><td>0.00</td><td>0.00</td><td>603</td><td>B:ALA 603</td><td></td><td>50.27</td><td>0.00</td><td>0.00</td></tr>
<tr><td>604</td><td>A:ALA 604</td><td></td><td>1.69</td><td>0.00</td><td>0.00</td><td>604</td><td>B:ALA 604</td><td></td><td>1.43</td><td>0.00</td><td>0.00</td></tr>
<tr><td>605</td><td>A:GLN 605</td><td></td><td>32.38</td><td>0.00</td><td>0.00</td><td>605</td><td>B:GLN 605</td><td></td><td>31.74</td><td>0.00</td><td>0.00</td></tr>
<tr><td>606</td><td>A:TYR 606</td><td></td><td>105.93</td><td>6.75 |</td><td>-0.08</td><td>606</td><td>B:TYR 606</td><td></td><td>104.04</td><td>7.49 |</td><td>-0.09</td></tr>
<tr><td>607</td><td>A:ARG 607</td><td></td><td>133.24</td><td>0.00</td><td>0.00</td><td>607</td><td>B:ARG 607</td><td></td><td>133.67</td><td>0.00</td><td>0.00</td></tr>
<tr><td>608</td><td>A:GLU 608</td><td></td><td>27.09</td><td>0.00</td><td>0.00</td><td>608</td><td>B:GLU 608</td><td></td><td>27.24</td><td>0.00</td><td>0.00</td></tr>
<tr><td>609</td><td>A:VAL 609</td><td></td><td>19.23</td><td>0.00</td><td>0.00</td><td>609</td><td>B:VAL 609</td><td></td><td>18.57</td><td>0.00</td><td>0.00</td></tr>
<tr><td>610</td><td>A:ARG 610</td><td></td><td>111.65</td><td>0.00</td><td>0.00</td><td>610</td><td>B:ARG 610</td><td></td><td>111.10</td><td>0.00</td><td>0.00</td></tr>
<tr><td>611</td><td>A:ASP 611</td><td></td><td>94.25</td><td>0.00</td><td>0.00</td><td>611</td><td>B:ASP 611</td><td></td><td>93.78</td><td>0.00</td><td>0.00</td></tr>
<tr><td>612</td><td>A:LEU 612</td><td></td><td>119.65</td><td>0.00</td><td>0.00</td><td>612</td><td>B:LEU 612</td><td></td><td>118.66</td><td>0.00</td><td>0.00</td></tr>
<tr><td>613</td><td>A:GLU 613</td><td></td><td>116.14</td><td>0.00</td><td>0.00</td><td>613</td><td>B:GLU 613</td><td></td><td>114.33</td><td>0.00</td><td>0.00</td></tr>
<tr><td>614</td><td>A:ARG 614</td><td></td><td>62.77</td><td>0.00</td><td>0.00</td><td>614</td><td>B:ARG 614</td><td></td><td>63.90</td><td>0.00</td><td>0.00</td></tr>
<tr><td>615</td><td>A:GLY 615</td><td></td><td>17.68</td><td>0.00</td><td>0.00</td><td>615</td><td>B:GLY 615</td><td></td><td>17.02</td><td>0.00</td><td>0.00</td></tr>
<tr><td>616</td><td>A:ILE 616</td><td></td><td>50.12</td><td>0.00</td><td>0.00</td><td>616</td><td>B:ILE 616</td><td></td><td>48.32</td><td>0.00</td><td>0.00</td></tr>
<tr><td>617</td><td>A:PRO 617</td><td></td><td>103.47</td><td>10.59 ||</td><td>0.13</td><td>617</td><td>B:PRO 617</td><td></td><td>103.16</td><td>10.82 ||</td><td>0.13</td></tr>
<tr><td>618</td><td>A:ASN 618</td><td>H</td><td>179.33</td><td>128.74 |||||||</td><td>-0.25</td><td>618</td><td>B:ASN 618</td><td>H</td><td>178.59</td><td>126.73 |||||||</td><td>-0.27</td></tr>
</table>

PDBe PISA v1.52 [20/10/2014]

is a member of
