# Supplementary material for: Characterization of the Corynebacterium glutamicum dehydroshikimate dehydratase QsuB and its potential for microbial production of protocatechuic acid
Source: PLoS One. 2020 Aug 21;15(8):e0231560. doi: 10.1371/journal.pone.0231560 (PMC7442255; doi:10.1371/journal.pone.0231560)
Supplement: S3 File — (PDF) [file pone.0231560.s005.pdf]

Protein Data Bank in Europe

PDBePISA

pdbe.org/pisa

Bringing Structure to Biology

PISA Interface.

interface # 1 in N-qsub_dim.pdb

interface #1/1 XML << < > >>

Interface Summary XML

	Structure 1		Structure 2	
**Selection range**	A		B	
**class**	Protein		Protein	
**symmetry operation**	x,y,z		,,	
**symmetry ID**	1_555		0_555	
**Number of atoms**				
**interface**	40	1.7%	39	1.6%
**surface**	1333	56.1%	1322	55.6%
**total**	2378	100.0%	2378	100.0%
**Number of residues**				
**interface**	11	3.7%	10	3.4%
**surface**	267	89.9%	268	90.2%
**total**	297	100.0%	297	100.0%
**Solvent-accessible area, Å**				
**interface**	297.3	2.2%	299.1	2.2%
**total**	13737.4	100.0%	13729.1	100.0%
**Solvation energy, kcal/mol**				
**isolated structure**	-239.2	100.0%	-239.1	100.0%
**gain on complex formation**	-0.4	0.2%	-0.6	0.3%
**average gain**	-1.8	0.8%	-1.8	0.8%
**P-value**	0.802		0.765	

View structure 1 | interface | structure 2

Download structure 1 | interface | structure 2

This interface scored

0.000

in Complex Formation Significance Score (CSS).

CSS ranges from 0 to 1 as interface relevance to complex formation increases.

Achieved CSS implies that the interface does not play any role in complex formation and seems to be a resul

Hydrogen bonds XML

##	Structure 1	Dist. [Å]	Structure 2
1	A:ARG 246[NH2]	3.73	B:GLU 249[OE1]
2	A:GLU 249[OE1]	3.82	B:ARG 246[NH2]

Salt bridges XML

##	Structure 1	Dist. [Å]	Structure 2
1	A:ARG 246[NH2]	3.73	B:GLU 249[OE1]
2	A:GLU 249[OE1]	3.82	B:ARG 246[NH2]

No disulfide bonds found

No covalent bonds found

Interfacing residues (not a contact table) XML Display level: Residues

Inaccessible residues | HSDC Residues making **H**ydrogen/**D**isulphide bond, **S**alt bridge or **C**ovalent link

Solvent-accessible residues | Interfacing residues

ASA Accessible Surface Area, Å² **BSA** Buried Surface Area, Å² **Δ^{i}G** Solvation energy effect, kcal/mol |||| Buried area percentage, one bar per 10%

##	Structure 1	HSDC	ASA	BSA	Δ^{i}G	##	Structure 2	HSDC	ASA	BSA	Δ^{i}G
1	A:MET 1		67.78	0.00	0.00	1	B:MET 1		68.83	0.00	0.00
2	A:ARG 2		83.69	0.00	0.00	2	B:ARG 2		83.46	0.00	0.00
3	A:THR 3		1.15	0.00	0.00	3	B:THR 3		0.61	0.00	0.00
4	A:SER 4		0.00	0.00	0.00	4	B:SER 4		0.00	0.00	0.00
5	A:ILE 5		1.62	0.00	0.00	5	B:ILE 5		1.87	0.00	0.00
6	A:ALA 6		0.00	0.00	0.00	6	B:ALA 6		0.17	0.00	0.00
7	A:THR 7		4.86	0.00	0.00	7	B:THR 7		3.52	0.00	0.00
8	A:VAL 8		15.35	0.00	0.00	8	B:VAL 8		14.64	0.00	0.00
9	A:CYS 9		0.00	0.00	0.00	9	B:CYS 9		0.00	0.00	0.00
10	A:LEU 10		4.21	0.00	0.00	10	B:LEU 10		4.33	0.00	0.00
11	A:SER 11		11.33	0.13 |	-0.00	11	B:SER 11		10.36	0.00	0.00
12	A:GLY 12		26.11	0.00	0.00	12	B:GLY 12		27.05	0.00	0.00
13	A:THR 13		85.99	0.00	0.00	13	B:THR 13		86.08	0.00	0.00
14	A:LEU 14		8.84	0.00	0.00	14	B:LEU 14		8.67	0.00	0.00
15	A:ALA 15		52.33	0.00	0.00	15	B:ALA 15		52.95	0.00	0.00
16	A:GLU 16		42.17	11.16 |||	-0.03	16	B:GLU 16		42.66	14.36 ||||	-0.01
17	A:LYS 17		10.74	0.00	0.00	17	B:LYS 17		10.69	0.00	0.00
18	A:LEU 18		1.17	0.00	0.00	18	B:LEU 18		1.17	0.00	0.00
19	A:ARG 19		106.35	51.20 |||||	-0.57	19	B:ARG 19		106.70	52.28 |||||	-0.37
20	A:ALA 20		0.49	0.00	0.00	20	B:ALA 20		0.62	0.00	0.00
21	A:ALA 21		1.67	0.00	0.00	21	B:ALA 21		1.67	0.00	0.00
22	A:ALA 22		28.98	0.00	0.00	22	B:ALA 22		28.50	0.00	0.00

23	A:ASP 23	90.15	0.00	0.00	23	B:ASP 23	92.04	0.00	0.00
24	A:ALA 24	10.78	0.00	0.00	24	B:ALA 24	10.05	0.00	0.00
25	A:GLY 25	43.98	0.00	0.00	25	B:GLY 25	43.33	0.00	0.00
26	A:PHE 26	10.02	0.00	0.00	26	B:PHE 26	10.36	0.00	0.00
27	A:ASP 27	43.98	0.00	0.00	27	B:ASP 27	44.85	0.00	0.00
28	A:GLY 28	0.00	0.00	0.00	28	B:GLY 28	0.00	0.00	0.00
29	A:VAL 29	1.68	0.00	0.00	29	B:VAL 29	1.51	0.00	0.00
30	A:GLU 30	0.00	0.00	0.00	30	B:GLU 30	0.00	0.00	0.00
31	A:ILE 31	0.00	0.00	0.00	31	B:ILE 31	0.00	0.00	0.00
32	A:PHE 32	27.80	0.00	0.00	32	B:PHE 32	27.31	0.00	0.00
33	A:GLU 33	44.01	0.00	0.00	33	B:GLU 33	43.04	0.00	0.00
34	A:GLN 34	98.98	0.00	0.00	34	B:GLN 34	97.95	0.00	0.00
35	A:ASP 35	20.25	0.00	0.00	35	B:ASP 35	20.63	0.00	0.00
36	A:LEU 36	20.83	0.00	0.00	36	B:LEU 36	20.61	0.00	0.00
37	A:VAL 37	113.99	0.00	0.00	37	B:VAL 37	113.23	0.00	0.00
38	A:VAL 38	111.01	0.00	0.00	38	B:VAL 38	109.27	0.00	0.00
39	A:SER 39	23.54	0.00	0.00	39	B:SER 39	24.31	0.00	0.00
40	A:PRO 40	136.02	0.00	0.00	40	B:PRO 40	136.19	0.00	0.00
41	A:HIS 41	90.23	0.00	0.00	41	B:HIS 41	90.83	0.00	0.00
42	A:SER 42	57.95	0.00	0.00	42	B:SER 42	57.39	0.00	0.00
43	A:ALA 43	13.11	0.00	0.00	43	B:ALA 43	13.23	0.00	0.00
44	A:GLU 44	62.63	0.00	0.00	44	B:GLU 44	62.71	0.00	0.00
45	A:GLN 45	67.21	0.00	0.00	45	B:GLN 45	67.52	0.00	0.00
46	A:ILE 46	1.47	0.00	0.00	46	B:ILE 46	1.49	0.00	0.00
47	A:ARG 47	68.95	0.00	0.00	47	B:ARG 47	69.37	0.00	0.00
48	A:GLN 48	60.52	0.00	0.00	48	B:GLN 48	59.87	0.00	0.00
49	A:ARG 49	59.02	0.00	0.00	49	B:ARG 49	58.30	0.00	0.00
50	A:ALA 50	4.57	0.00	0.00	50	B:ALA 50	4.75	0.00	0.00
51	A:GLN 51	108.48	0.00	0.00	51	B:GLN 51	107.32	0.00	0.00
52	A:ASP 52	89.31	0.00	0.00	52	B:ASP 52	90.84	0.00	0.00
53	A:LEU 53	72.91	0.00	0.00	53	B:LEU 53	73.15	0.00	0.00
54	A:GLU 54	166.73	0.00	0.00	54	B:GLU 54	167.26	0.00	0.00
55	A:LEU 55	4.26	0.00	0.00	55	B:LEU 55	4.50	0.00	0.00
56	A:THR 56	63.42	0.00	0.00	56	B:THR 56	63.59	0.00	0.00
57	A:LEU 57	9.84	0.00	0.00	57	B:LEU 57	9.38	0.00	0.00
58	A:ASP 58	21.64	0.00	0.00	58	B:ASP 58	21.53	0.00	0.00
59	A:LEU 59	0.00	0.00	0.00	59	B:LEU 59	0.00	0.00	0.00
60	A:PHE 60	0.31	0.00	0.00	60	B:PHE 60	0.16	0.00	0.00
61	A:GLN 61	4.07	0.00	0.00	61	B:GLN 61	4.37	0.00	0.00
62	A:PRO 62	32.55	0.00	0.00	62	B:PRO 62	33.07	0.00	0.00
63	A:PHE 63	1.25	0.00	0.00	63	B:PHE 63	1.39	0.00	0.00
64	A:ARG 64	71.43	0.00	0.00	64	B:ARG 64	71.34	0.00	0.00
65	A:ASP 65	41.08	0.00	0.00	65	B:ASP 65	42.73	0.00	0.00
66	A:PHE 66	0.28	0.00	0.00	66	B:PHE 66	0.12	0.00	0.00
67	A:GLU 67	0.00	0.00	0.00	67	B:GLU 67	0.00	0.00	0.00
68	A:GLY 68	0.17	0.00	0.00	68	B:GLY 68	0.17	0.00	0.00
69	A:VAL 69	24.21	0.00	0.00	69	B:VAL 69	24.69	0.00	0.00
70	A:GLU 70	78.35	0.00	0.00	70	B:GLU 70	78.62	0.00	0.00
71	A:GLU 71	130.71	0.00	0.00	71	B:GLU 71	130.51	0.00	0.00
72	A:GLU 72	99.60	0.00	0.00	72	B:GLU 72	99.57	0.00	0.00
73	A:GLN 73	78.76	0.00	0.00	73	B:GLN 73	78.54	0.00	0.00
74	A:PHE 74	35.76	0.00	0.00	74	B:PHE 74	35.58	0.00	0.00
75	A:LEU 75	115.36	0.00	0.00	75	B:LEU 75	115.82	0.00	0.00
76	A:LYS 76	110.25	0.00	0.00	76	B:LYS 76	109.83	0.00	0.00
77	A:ASN 77	10.12	0.00	0.00	77	B:ASN 77	9.49	0.00	0.00
78	A:LEU 78	33.41	0.00	0.00	78	B:LEU 78	32.41	0.00	0.00
79	A:HIS 79	110.79	0.00	0.00	79	B:HIS 79	110.74	0.00	0.00
80	A:ARG 80	99.79	0.00	0.00	80	B:ARG 80	97.52	0.00	0.00
81	A:LEU 81	0.50	0.00	0.00	81	B:LEU 81	0.17	0.00	0.00
82	A:GLU 82	38.71	0.00	0.00	82	B:GLU 82	37.97	0.00	0.00
83	A:GLU 83	78.33	0.00	0.00	83	B:GLU 83	78.63	0.00	0.00
84	A:LYS 84	14.81	0.00	0.00	84	B:LYS 84	15.17	0.00	0.00
85	A:PHE 85	0.17	0.00	0.00	85	B:PHE 85	0.00	0.00	0.00
86	A:LYS 86	68.16	0.00	0.00	86	B:LYS 86	68.11	0.00	0.00
87	A:LEU 87	44.72	0.00	0.00	87	B:LEU 87	44.74	0.00	0.00
88	A:MET 88	0.00	0.00	0.00	88	B:MET 88	0.00	0.00	0.00
89	A:ASN 89	56.96	0.00	0.00	89	B:ASN 89	56.83	0.00	0.00
90	A:ARG 90	134.03	0.00	0.00	90	B:ARG 90	131.88	0.00	0.00
91	A:LEU 91	2.33	0.00	0.00	91	B:LEU 91	2.33	0.00	0.00
92	A:GLY 92	63.30	0.00	0.00	92	B:GLY 92	62.79	0.00	0.00
93	A:ILE 93	7.91	0.00	0.00	93	B:ILE 93	8.73	0.00	0.00
94	A:GLU 94	72.71	0.00	0.00	94	B:GLU 94	74.58	0.00	0.00
95	A:MET 95	20.83	0.00	0.00	95	B:MET 95	20.00	0.00	0.00
96	A:ILE 96	0.99	0.00	0.00	96	B:ILE 96	0.66	0.00	0.00
97	A:LEU 97	4.52	0.00	0.00	97	B:LEU 97	4.86	0.00	0.00
98	A:LEU 98	0.83	0.00	0.00	98	B:LEU 98	1.00	0.00	0.00
99	A:CYS 99	0.00	0.00	0.00	99	B:CYS 99	0.00	0.00	0.00
100	A:SER 100	0.00	0.00	0.00	100	B:SER 100	0.12	0.00	0.00
101	A:ASN 101	0.73	0.00	0.00	101	B:ASN 101	1.04	0.00	0.00
102	A:VAL 102	54.48	0.00	0.00	102	B:VAL 102	54.14	0.00	0.00
103	A:GLY 103	27.72	0.00	0.00	103	B:GLY 103	28.20	0.00	0.00

104	A:THR 104	125.39	0.00	0.00	104	B:THR 104	125.77	0.00	0.00
105	A:ALA 105	74.25	0.00	0.00	105	B:ALA 105	76.23	0.00	0.00
106	A:THR 106	4.55	0.00	0.00	106	B:THR 106	4.30	0.00	0.00
107	A:ILE 107	73.78	0.00	0.00	107	B:ILE 107	73.69	0.00	0.00
108	A:ASN 108	81.96	0.00	0.00	108	B:ASN 108	81.52	0.00	0.00
109	A:ASP 109	73.03	0.00	0.00	109	B:ASP 109	72.58	0.00	0.00
110	A:ASP 110	57.86	0.00	0.00	110	B:ASP 110	57.84	0.00	0.00
111	A:ASP 111	83.93	0.00	0.00	111	B:ASP 111	83.43	0.00	0.00
112	A:LEU 112	61.61	0.00	0.00	112	B:LEU 112	58.94	0.00	0.00
113	A:PHE 113	0.16	0.00	0.00	113	B:PHE 113	0.31	0.00	0.00
114	A:VAL 114	20.19	0.00	0.00	114	B:VAL 114	20.35	0.00	0.00
115	A:GLU 115	72.84	0.00	0.00	115	B:GLU 115	72.42	0.00	0.00
116	A:GLN 116	16.98	0.00	0.00	116	B:GLN 116	15.91	0.00	0.00
117	A:LEU 117	0.00	0.00	0.00	117	B:LEU 117	0.17	0.00	0.00
118	A:HIS 118	44.78	0.00	0.00	118	B:HIS 118	45.36	0.00	0.00
119	A:ARG 119	95.04	0.00	0.00	119	B:ARG 119	93.30	0.00	0.00
120	A:ALA 120	0.33	0.00	0.00	120	B:ALA 120	0.33	0.00	0.00
121	A:ALA 121	0.00	0.00	0.00	121	B:ALA 121	0.00	0.00	0.00
122	A:ASP 122	46.43	0.00	0.00	122	B:ASP 122	45.67	0.00	0.00
123	A:LEU 123	44.76	0.00	0.00	123	B:LEU 123	45.44	0.00	0.00
124	A:ALA 124	0.00	0.00	0.00	124	B:ALA 124	0.00	0.00	0.00
125	A:GLU 125	76.74	0.00	0.00	125	B:GLU 125	75.61	0.00	0.00
126	A:LYS 126	112.69	0.00	0.00	126	B:LYS 126	110.76	0.00	0.00
127	A:TYR 127	96.88	0.00	0.00	127	B:TYR 127	95.66	0.00	0.00
128	A:ASN 128	97.06	0.00	0.00	128	B:ASN 128	97.95	0.00	0.00
129	A:VAL 129	1.00	0.00	0.00	129	B:VAL 129	1.34	0.00	0.00
130	A:LYS 130	58.06	0.00	0.00	130	B:LYS 130	57.55	0.00	0.00
131	A:ILE 131	1.50	0.00	0.00	131	B:ILE 131	1.17	0.00	0.00
132	A:ALA 132	0.00	0.00	0.00	132	B:ALA 132	0.00	0.00	0.00
133	A:TYR 133	0.00	0.00	0.00	133	B:TYR 133	0.00	0.00	0.00
134	A:GLU 134	5.08	0.00	0.00	134	B:GLU 134	5.10	0.00	0.00
135	A:ALA 135	6.05	0.00	0.00	135	B:ALA 135	6.46	0.00	0.00
136	A:LEU 136	10.59	0.00	0.00	136	B:LEU 136	11.25	0.00	0.00
137	A:ALA 137	5.82	0.00	0.00	137	B:ALA 137	5.69	0.00	0.00
138	A:TRP 138	113.30	0.00	0.00	138	B:TRP 138	112.54	0.00	0.00
139	A:GLY 139	2.37	0.00	0.00	139	B:GLY 139	2.89	0.00	0.00
140	A:LYS 140	86.42	0.00	0.00	140	B:LYS 140	85.74	0.00	0.00
141	A:PHE 141	56.32	0.00	0.00	141	B:PHE 141	56.97	0.00	0.00
142	A:VAL 142	0.82	0.00	0.00	142	B:VAL 142	0.80	0.00	0.00
143	A:ASN 143	33.27	0.00	0.00	143	B:ASN 143	33.52	0.00	0.00
144	A:ASP 144	3.27	0.00	0.00	144	B:ASP 144	3.14	0.00	0.00
145	A:PHE 145	3.21	0.00	0.00	145	B:PHE 145	3.56	0.00	0.00
146	A:GLU 146	62.97	0.00	0.00	146	B:GLU 146	62.77	0.00	0.00
147	A:HIS 147	55.55	0.00	0.00	147	B:HIS 147	55.37	0.00	0.00
148	A:ALA 148	0.00	0.00	0.00	148	B:ALA 148	0.00	0.00	0.00
149	A:HIS 149	42.14	0.00	0.00	149	B:HIS 149	41.73	0.00	0.00
150	A:ALA 150	58.07	0.00	0.00	150	B:ALA 150	57.49	0.00	0.00
151	A:LEU 151	7.21	0.00	0.00	151	B:LEU 151	6.88	0.00	0.00
152	A:VAL 152	0.50	0.00	0.00	152	B:VAL 152	0.83	0.00	0.00
153	A:GLU 153	50.11	0.00	0.00	153	B:GLU 153	50.68	0.00	0.00
154	A:LYS 154	99.97	0.00	0.00	154	B:LYS 154	99.42	0.00	0.00
155	A:VAL 155	0.50	0.00	0.00	155	B:VAL 155	0.17	0.00	0.00
156	A:ASN 156	96.39	0.00	0.00	156	B:ASN 156	96.37	0.00	0.00
157	A:HIS 157	8.19	0.00	0.00	157	B:HIS 157	9.17	0.00	0.00
158	A:LYS 158	138.42	0.00	0.00	158	B:LYS 158	138.22	0.00	0.00
159	A:ALA 159	3.51	0.00	0.00	159	B:ALA 159	3.34	0.00	0.00
160	A:LEU 160	0.12	0.00	0.00	160	B:LEU 160	0.12	0.00	0.00
161	A:GLY 161	0.00	0.00	0.00	161	B:GLY 161	0.00	0.00	0.00
162	A:THR 162	0.80	0.00	0.00	162	B:THR 162	0.91	0.00	0.00
163	A:CYS 163	0.45	0.00	0.00	163	B:CYS 163	0.66	0.00	0.00
164	A:LEU 164	1.00	0.00	0.00	164	B:LEU 164	1.00	0.00	0.00
165	A:ASP 165	2.81	0.00	0.00	165	B:ASP 165	3.27	0.00	0.00
166	A:THR 166	0.00	0.00	0.00	166	B:THR 166	0.00	0.00	0.00
167	A:PHE 167	0.00	0.00	0.00	167	B:PHE 167	0.00	0.00	0.00
168	A:HIS 168	15.54	0.00	0.00	168	B:HIS 168	15.04	0.00	0.00
169	A:ILE 169	5.85	0.00	0.00	169	B:ILE 169	6.35	0.00	0.00
170	A:LEU 170	12.29	0.00	0.00	170	B:LEU 170	11.22	0.00	0.00
171	A:SER 171	9.18	0.00	0.00	171	B:SER 171	9.35	0.00	0.00
172	A:ARG 172	79.17	0.00	0.00	172	B:ARG 172	80.35	0.00	0.00
173	A:GLY 173	63.01	0.00	0.00	173	B:GLY 173	64.08	0.00	0.00
174	A:TRP 174	112.73	0.00	0.00	174	B:TRP 174	111.02	0.00	0.00
175	A:GLU 175	89.63	0.00	0.00	175	B:GLU 175	89.22	0.00	0.00
176	A:THR 176	5.07	0.00	0.00	176	B:THR 176	4.36	0.00	0.00
177	A:ASP 177	65.41	0.00	0.00	177	B:ASP 177	64.80	0.00	0.00
178	A:GLU 178	79.50	0.00	0.00	178	B:GLU 178	78.80	0.00	0.00
179	A:VAL 179	2.01	0.00	0.00	179	B:VAL 179	1.50	0.00	0.00
180	A:GLU 180	46.06	0.00	0.00	180	B:GLU 180	44.66	0.00	0.00
181	A:ASN 181	102.42	0.00	0.00	181	B:ASN 181	101.40	0.00	0.00
182	A:ILE 182	2.95	0.00	0.00	182	B:ILE 182	2.51	0.00	0.00
183	A:PRO 183	64.50	0.00	0.00	183	B:PRO 183	63.17	0.00	0.00
184	A:ALA 184	18.42	0.00	0.00	184	B:ALA 184	18.14	0.00	0.00

185	A:GLU 185		137.41	0.00		0.00	185	B:GLU 185		137.65	0.00		0.00
186	A:LYS 186		24.58	0.00		0.00	186	B:LYS 186		25.01	0.00		0.00
187	A:ILE 187		0.00	0.00		0.00	187	B:ILE 187		0.00	0.00		0.00
188	A:PHE 188		37.82	0.00		0.00	188	B:PHE 188		38.03	0.00		0.00
189	A:PHE 189		0.16	0.00		0.00	189	B:PHE 189		0.00	0.00		0.00
190	A:VAL 190		0.00	0.00		0.00	190	B:VAL 190		0.00	0.00		0.00
191	A:GLN 191		4.35	0.00		0.00	191	B:GLN 191		4.89	0.00		0.00
192	A:LEU 192		0.00	0.00		0.00	192	B:LEU 192		0.00	0.00		0.00
193	A:ALA 193		0.00	0.00		0.00	193	B:ALA 193		0.00	0.00		0.00
194	A:ASP 194		1.97	0.00		0.00	194	B:ASP 194		1.96	0.00		0.00
195	A:ALA 195		0.00	0.00		0.00	195	B:ALA 195		0.00	0.00		0.00
196	A:PRO 196		52.11	0.00		0.00	196	B:PRO 196		51.43	0.00		0.00
197	A:LYS 197		100.57	0.00		0.00	197	B:LYS 197		100.33	0.00		0.00
198	A:LEU 198		164.50	0.00		0.00	198	B:LEU 198		166.58	0.00		0.00
199	A:SER 199		37.21	0.00		0.00	199	B:SER 199		37.58	0.00		0.00
200	A:MET 200		166.02	0.00		0.00	200	B:MET 200		166.29	0.00		0.00
201	A:ASP 201		83.13	0.00		0.00	201	B:ASP 201		84.90	0.00		0.00
202	A:ILE 202		28.29	0.00		0.00	202	B:ILE 202		28.44	0.00		0.00
203	A:LEU 203		77.30	0.00		0.00	203	B:LEU 203		77.05	0.00		0.00
204	A:SER 204		52.75	0.00		0.00	204	B:SER 204		52.28	0.00		0.00
205	A:TRP 205		23.91	0.00		0.00	205	B:TRP 205		24.22	0.00		0.00
206	A:SER 206		17.25	0.00		0.00	206	B:SER 206		16.76	0.00		0.00
207	A:ARG 207		78.64	0.00		0.00	207	B:ARG 207		79.52	0.00		0.00
208	A:HIS 208		127.83	0.00		0.00	208	B:HIS 208		129.46	0.00		0.00
209	A:HIS 209		54.76	0.00		0.00	209	B:HIS 209		56.71	0.00		0.00
210	A:ARG 210		22.40	0.00		0.00	210	B:ARG 210		21.99	0.00		0.00
211	A:VAL 211		41.73	0.00		0.00	211	B:VAL 211		42.24	0.00		0.00
212	A:PHE 212		4.08	0.00		0.00	212	B:PHE 212		3.92	0.00		0.00
213	A:PRO 213		8.85	0.00		0.00	213	B:PRO 213		9.00	0.00		0.00
214	A:GLY 214		67.12	0.00		0.00	214	B:GLY 214		67.43	0.00		0.00
215	A:GLU 215		105.31	0.00		0.00	215	B:GLU 215		106.87	0.00		0.00
216	A:GLY 216		32.51	0.00		0.00	216	B:GLY 216		31.67	0.00		0.00
217	A:ASP 217		71.00	0.00		0.00	217	B:ASP 217		70.35	0.00		0.00
218	A:PHE 218		35.77	0.00		0.00	218	B:PHE 218		35.79	0.00		0.00
219	A:ASP 219		67.79	0.00		0.00	219	B:ASP 219		68.64	0.00		0.00
220	A:LEU 220		4.98	0.00		0.00	220	B:LEU 220		5.44	0.00		0.00
221	A:VAL 221		40.02	0.00		0.00	221	B:VAL 221		40.48	0.00		0.00
222	A:LYS 222		61.16	0.00		0.00	222	B:LYS 222		61.15	0.00		0.00
223	A:PHE 223		3.04	0.00		0.00	223	B:PHE 223		2.81	0.00		0.00
224	A:MET 224		2.84	0.00		0.00	224	B:MET 224		2.51	0.00		0.00
225	A:VAL 225		5.35	0.00		0.00	225	B:VAL 225		5.68	0.00		0.00
226	A:HIS 226		7.53	0.00		0.00	226	B:HIS 226		7.06	0.00		0.00
227	A:LEU 227		0.17	0.00		0.00	227	B:LEU 227		0.33	0.00		0.00
228	A:ALA 228		22.00	0.00		0.00	228	B:ALA 228		22.29	0.00		0.00
229	A:LYS 229		38.90	0.00		0.00	229	B:LYS 229		37.79	0.00		0.00
230	A:THR 230		28.02	0.00		0.00	230	B:THR 230		27.72	0.00		0.00
231	A:GLY 231		51.19	0.00		0.00	231	B:GLY 231		51.94	0.00		0.00
232	A:TYR 232		14.96	0.00		0.00	232	B:TYR 232		15.47	0.00		0.00
233	A:ASP 233		100.11	0.00		0.00	233	B:ASP 233		99.00	0.00		0.00
234	A:GLY 234		14.52	0.00		0.00	234	B:GLY 234		15.06	0.00		0.00
235	A:PRO 235		26.26	0.00		0.00	235	B:PRO 235		25.60	0.00		0.00
236	A:ILE 236		2.84	0.00		0.00	236	B:ILE 236		2.01	0.00		0.00
237	A:SER 237		0.00	0.00		0.00	237	B:SER 237		0.00	0.00		0.00
238	A:LEU 238		0.00	0.00		0.00	238	B:LEU 238		0.00	0.00		0.00
239	A:GLU 239		13.56	0.00		0.00	239	B:GLU 239		14.18	0.00		0.00
240	A:ILE 240		17.42	0.00		0.00	240	B:ILE 240		17.51	0.00		0.00
241	A:PHE 241		78.19	0.00		0.00	241	B:PHE 241		78.01	0.00		0.00
242	A:ASN 242		26.97	0.00		0.00	242	B:ASN 242		27.22	0.00		0.00
243	A:ASP 243		47.15	0.00		0.00	243	B:ASP 243		47.08	0.00		0.00
244	A:SER 244		114.43	0.00		0.00	244	B:SER 244		113.36	0.00		0.00
245	A:PHE 245		161.00	0.00		0.00	245	B:PHE 245		160.21	0.00		0.00
246	A:ARG 246	HS	142.29	62.69	|||||	-0.68	246	B:ARG 246	HS	142.97	60.86	|||||	-0.65
247	A:LYS 247		86.35	7.88	|	0.11	247	B:LYS 247		86.37	6.15	|	0.08
248	A:ALA 248		30.09	6.75	|||	-0.05	248	B:ALA 248		30.64	6.97	|||	-0.04
249	A:GLU 249	HS	102.06	97.31	||||||||||	0.74	249	B:GLU 249	HS	103.34	99.58	||||||||||	0.72
250	A:VAL 250		3.06	2.56	|||||||||	0.04	250	B:VAL 250		3.09	2.42	||||||||	0.04
251	A:GLY 251		10.99	3.50	||||	0.06	251	B:GLY 251		10.35	3.51	||||	0.06
252	A:ARG 252		105.25	46.83	|||||	0.69	252	B:ARG 252		104.18	44.99	|||||	0.67
253	A:THR 253		3.01	0.00		0.00	253	B:THR 253		2.68	0.00		0.00
254	A:ALA 254		0.00	0.00		0.00	254	B:ALA 254		0.00	0.00		0.00
255	A:ILE 255		71.07	7.29	||	0.12	255	B:ILE 255		73.02	7.96	||	0.13
256	A:ASP 256		32.20	0.00		0.00	256	B:ASP 256		30.53	0.00		0.00
257	A:GLY 257		0.00	0.00		0.00	257	B:GLY 257		0.00	0.00		0.00
258	A:LEU 258		28.60	0.00		0.00	258	B:LEU 258		28.92	0.00		0.00
259	A:ARG 259		138.32	0.00		0.00	259	B:ARG 259		135.88	0.00		0.00
260	A:SER 260		10.69	0.00		0.00	260	B:SER 260		10.91	0.00		0.00
261	A:LEU 261		0.12	0.00		0.00	261	B:LEU 261		0.12	0.00		0.00
262	A:ARG 262		73.89	0.00		0.00	262	B:ARG 262		74.64	0.00		0.00
263	A:TRP 263		137.11	0.00		0.00	263	B:TRP 263		141.79	0.00		0.00
264	A:LEU 264		7.47	0.00		0.00	264	B:LEU 264		7.15	0.00		0.00
265	A:GLU 265		6.14	0.00		0.00	265	B:GLU 265		6.56	0.00		0.00

266	A:ASP 266	31.91	0.00	0.00	266	B:ASP 266	31.56	0.00	0.00
267	A:GLN 267	80.15	0.00	0.00	267	B:GLN 267	81.77	0.00	0.00
268	A:THR 268	0.00	0.00	0.00	268	B:THR 268	0.00	0.00	0.00
269	A:TRP 269	45.49	0.00	0.00	269	B:TRP 269	44.88	0.00	0.00
270	A:HIS 270	134.23	0.00	0.00	270	B:HIS 270	132.64	0.00	0.00
271	A:ALA 271	29.09	0.00	0.00	271	B:ALA 271	28.75	0.00	0.00
272	A:LEU 272	3.19	0.00	0.00	272	B:LEU 272	3.19	0.00	0.00
273	A:ASN 273	75.56	0.00	0.00	273	B:ASN 273	75.61	0.00	0.00
274	A:ALA 274	91.19	0.00	0.00	274	B:ALA 274	90.12	0.00	0.00
275	A:GLU 275	63.77	0.00	0.00	275	B:GLU 275	64.23	0.00	0.00
276	A:ASP 276	118.89	0.00	0.00	276	B:ASP 276	118.25	0.00	0.00
277	A:ARG 277	107.83	0.00	0.00	277	B:ARG 277	108.69	0.00	0.00
278	A:PRO 278	77.08	0.00	0.00	278	B:PRO 278	77.16	0.00	0.00
279	A:SER 279	38.63	0.00	0.00	279	B:SER 279	38.58	0.00	0.00
280	A:ALA 280	71.93	0.00	0.00	280	B:ALA 280	72.66	0.00	0.00
281	A:LEU 281	108.02	0.00	0.00	281	B:LEU 281	106.41	0.00	0.00
282	A:GLU 282	146.79	0.00	0.00	282	B:GLU 282	147.57	0.00	0.00
283	A:LEU 283	79.60	0.00	0.00	283	B:LEU 283	78.58	0.00	0.00
284	A:ARG 284	11.00	0.00	0.00	284	B:ARG 284	11.09	0.00	0.00
285	A:ALA 285	27.86	0.00	0.00	285	B:ALA 285	29.46	0.00	0.00
286	A:LEU 286	90.11	0.00	0.00	286	B:LEU 286	90.77	0.00	0.00
287	A:PRO 287	66.47	0.00	0.00	287	B:PRO 287	67.41	0.00	0.00
288	A:GLU 288	139.88	0.00	0.00	288	B:GLU 288	140.36	0.00	0.00
289	A:VAL 289	114.48	0.00	0.00	289	B:VAL 289	113.78	0.00	0.00
290	A:ALA 290	84.10	0.00	0.00	290	B:ALA 290	83.53	0.00	0.00
291	A:GLU 291	158.83	0.00	0.00	291	B:GLU 291	159.73	0.00	0.00
292	A:PRO 292	108.04	0.00	0.00	292	B:PRO 292	107.88	0.00	0.00
293	A:GLU 293	190.33	0.00	0.00	293	B:GLU 293	189.75	0.00	0.00
294	A:GLY 294	52.26	0.00	0.00	294	B:GLY 294	51.82	0.00	0.00
295	A:VAL 295	141.53	0.00	0.00	295	B:VAL 295	142.21	0.00	0.00
296	A:ASP 296	120.05	0.00	0.00	296	B:ASP 296	121.55	0.00	0.00
297	A:PHE 297	234.82	0.00	0.00	297	B:PHE 297	232.53	0.00	0.00

PDBe PISA v1.52 [20/10/2014]

PDBe Protein Data Bank in Europe

is a member of

PDB

EMDataBank Unified Data Resource for 3DEM
